# Stripenn detects architectural stripes from chromatin conformation data using computer vision

**DOI:** 10.1038/s41467-022-29258-9

**Published:** 2022-03-24

**Authors:** Sora Yoon, Aditi Chandra, Golnaz Vahedi

**Affiliations:** 1 Department of Genetics, Pennsylvania Perelman School of Medicine, Philadelphia, PA 19104 USA; 2 Institute for Immunology, Pennsylvania Perelman School of Medicine, Philadelphia, PA 19104 USA; 3 Epigenetics Institute, Pennsylvania Perelman School of Medicine, Philadelphia, PA 19104 USA; 4 Institute for Diabetes, Obesity and Metabolism, Pennsylvania Perelman School of Medicine, Philadelphia, PA 19104 USA; 5 Abramson Family Cancer Research Institute, University of Pennsylvania Perelman School of Medicine, Philadelphia, PA 19104 USA

**Keywords:** Software, Epigenomics

## Abstract

Architectural stripes tend to form at genomic regions harboring genes with salient roles in cell identity and function. Therefore, the accurate identification and quantification of these features are essential for understanding lineage-specific gene regulation. Here, we present Stripenn, an algorithm rooted in computer vision to systematically detect and quantitate architectural stripes from chromatin conformation measurements using various technologies. We demonstrate that Stripenn outperforms existing methods and highlight its biological applications in the context of B and T lymphocytes. By comparing stripes across distinct cell types and different species, we find that these chromatin features are highly conserved and form at genes with prominent roles in cell-type-specific processes. In summary, Stripenn is a computational method that borrows concepts from widely used image processing techniques to demarcate and quantify architectural stripes.

## Abstract (web summary)

Chromosome conformation capture techniques have recently revealed features beyond chromatin loops such as architectural stripes. Here the authors present their stripe detection tool ‘Stripenn’ to detect and quantitate stripes from any type of chromatin conformation capture data. They show that architectural stripes are enriched at transcriptionally active and accessible genomic regions.

## Introduction

The eukaryotic genome is tightly organized inside the nucleus by forming a complex of DNA and histone proteins called the chromatin^1–3^. Chromosome conformation capture techniques, in particular Hi-C, suggest that the chromatin is folded into various length scales, forming a hierarchical structure^4,5^. Among these structures, topologically associating domains (TADs) are sub-megabase regions where stronger interactions are observed between loci inside each domain compared with loci in neighboring domains^6,7^. The spatial proximity of two genomic regions within TADs displaying high contact frequency in Hi-C maps is referred to as chromatin loops^8,9^. Loop formation, which is best described by the loop extrusion model, is mediated through sliding cohesin anchored by CTCF binding events in a convergent orientation^10,11^.

Although TADs and chromatin loops as distinguishing features of Hi-C maps were described in pioneering studies, structures appearing as lines, flames or stripes attracted attention most recently^10,12–14^. Architectural stripes are thought to form through the loop extrusion process when a loop anchor interacts with the entire domain at high frequency^13^. Stripes have been reported as frequent features of diverse developmental programs^15^. It has been proposed that these unique structures, often associated with active enhancers and super-enhancers, can tether enhancers to cognate promoters, facilitating transcription and recombination^13^. Moreover, stripe anchors represent major hotspots for topoisomerase-mediated lesions, which may promote chromosomal translocations and cancer^13^. Since architectural stripes tend to form at genomic regions harboring genes with key roles in cell identity and function, it is essential to accurately detect these features from Hi-C or other chromatin conformation capture measurements^12,13,16^.

Numerous computational techniques have been developed to detect chromatin loops. Yet, the reliable identification of architectural stripes remains a challenge^17–21^. The first reported stripe detection algorithm, referred to as Zebra, exploited the Poisson statistics and yielded thousands of stripes^13^. Although the original study reporting Zebra detects regions with stripy features, the algorithm suffers from three major limitations: (a) it has a high false-positive rate and detects some chromatin loops as stripes, (b) it lacks a quantitative assessment of stripes and has been reported to rely on manual curation, and (c) the code for the algorithm’s implementation in the original study^13^ is not publicly available. Recently, an implementation of the Zebra algorithm, referred to as StripeCaller^22^, was made available on Github by an independent group. However, the accuracy and quality of stripes detected by this implementation, which is not peer-reviewed, remains unknown.

Another computational approach, which is publicly available and referred to as domainClassifyR, was developed to comprehensively detect stripes and loops by first marking TADs and then measuring their stripe and loop scores^12^. However, domainClassifyR assumes that stripes form exclusively at the boundaries of genomic domains defined by TAD callers. It is evident from Hi-C maps of diverse cell types that although some architectural stripes form at TAD boundaries, stripy features are also found inside TADs. Hence, intra-TAD stripes remain undetected by the domainClassifyR computational approach. Another tool called CHESS was recently developed to perform quantitative comparisons of chromatin contact data between two conditions using the structural similarity index^23^. Although CHESS has been developed to report differential features such as TADs, stripes, or loops between two conditions^23^, this method cannot be used to delineate architectural stripes in a cell type of interest. Another relevant method is Chromosight, which was developed to detect specific patterns such as loop and hairpin structures using computer vision^21^. Technically, Chromosight can also be applicable in discovering stripes using appropriate kernels. However, it remains unclear if this technique can extract stripe coordinates which is important for downstream biologically relevant investigations. Altogether, despite the availability of multiple computational techniques, there is a need to accurately and efficiently detect architectural stripes and assess the strength of stripes across cell types and conditions.

Here, we report the development of a specialized stripe detection tool called Stripenn, which borrows concepts from computer vision and image processing. The backbone of our method relies on Canny edge detection^24^, one of the most popular edge detection algorithms in the mature field of computer vision. Stripenn can be applied to any chromatin conformation capture data such as Hi-C^4^, HiChIP^25^, and Micro-C^26^. Our method offers two scoring systems: *P*-value to filter low-quality stripes and *stripiness* to rank stripes based on the continuity of interaction signal. Here we show that Stripenn outperforms existing techniques including Zebra, Chromosight, and domainClassifyR when these techniques are applied on the same ultra-high coverage Hi-C dataset. Our systematic analysis of stripes from B and T lymphocytes using Stripenn indicates that the majority of stripes are on the transcriptionally active compartment. Moreover, cohesin loader NIPBL and active enhancer modifications are highly enriched at stripe domains, and active enhancers are biased to only one or few genes within stripes. Although hundreds of genes are differentially expressed between the two inbred mouse strains, most stripes are conserved and demonstrate comparable stripiness. In contrast, the comparison of B and T lymphocytes shows a large number of cell-type-specific stripes corresponding to cell-type-specific gene expression. Finally, the conserved stripes across human and mouse T cells harbor significantly more genes related to T cell biology than those in the conserved loops. Together, Stripenn, which is freely available on Github^27^, is a specialized tool dedicated for stripe detection, enabling the systematic and quantitative analysis of stripes.

## Results

### Overview of the Stripenn algorithm

**Fig. 1:**
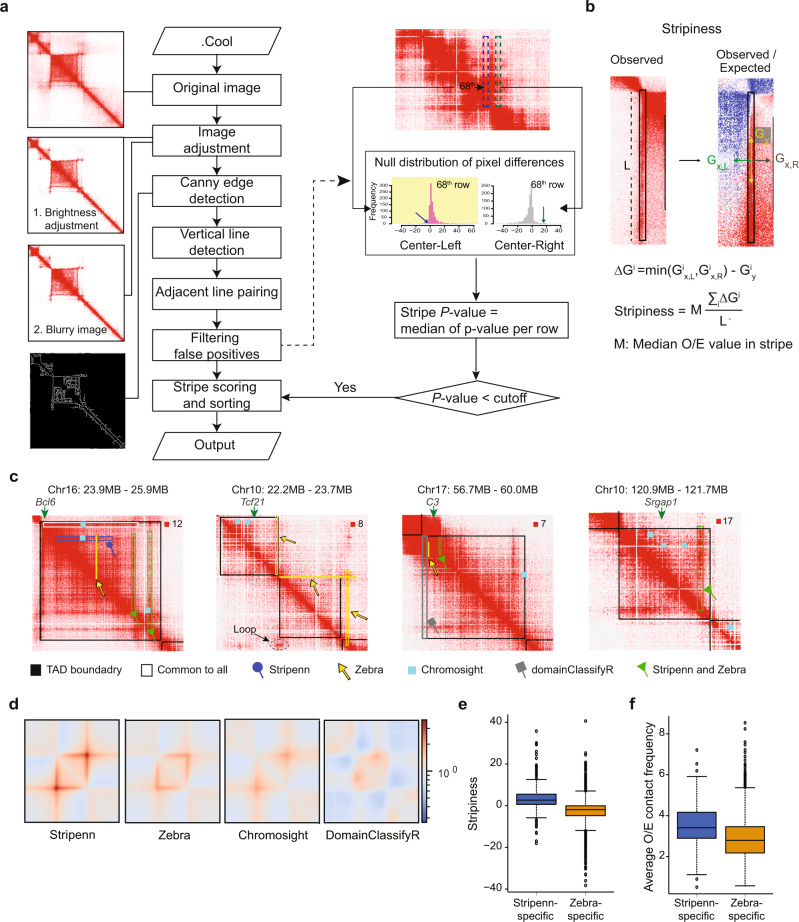
Stripenn overview and comparison with existing stripe callers. **a** Stripenn searches for candidate stripes using image-processing techniques (left) and filters them based on median *P*-value calculation which estimates the contrast of stripes and corresponding backgrounds (right). *P*-value is estimated for every row in a (vertical) stripe and the median is used as a median *P*-value. For each row, *P*-value is estimated based on the pixel difference within the stripe and its left- or right-adjacent background (blue and green dashed border, respectively). Among two *P*-values estimated from left and right backgrounds, the larger *P*-value is chosen (yellow box). Since the expected contact frequency decreases as the genomic distance of two DNA regions increases, different null distribution is used for each row. A mock example of the 68th row is provided. **b** Filtered stripes are further ranked using stripiness measured based on the observed/expected (O/E) contact frequency matrix. ${G}_{y}^{i}$: gradient in *y*-direction of i^th^ row. ${G}_{x,L}^{i}$ and ${G}_{x,R}^{i}$: gradient in *x*-direction of i^th^ row (left and right direction, respectively): $\triangle {G}^{i}$ refers to the difference of gradient in y-direction and x-direction. *L* refers to the length of the stripe. **c** Representative stripes detected by Stripenn, Zebra, Chromosight and/or domainClassifyR are demarcated on Hi-C contact matrices. Black borders represent TAD boundary. **d** Pileup plots demonstrate the quality of stripes detected by Stripenn, Zebra, Chromosight and domainClassifyR. **e**, **f** The distributions of stripiness (**e**) and the average observed/expected (O/E) contact frequency (**f**) of Stripenn-specific (*N* = 254 stripes) and Zebra-specific stripes (*N* = 3767 stripes). Data are shown as boxplots (centre, median; box limits, upper (75th) and lower (25th) percentiles; whiskers, 1.5× interquartile range; points, outliers).

Stripenn systematically detects and quantitates architectural stripes from genome-wide chromatin conformation measurements. Our method applies principles from computer vision to detect genomic anchors that interact with entire domains at high frequencies (Fig. 1a). The program’s output is a table containing coordinates and scores of predicted stripes, which can be used for downstream analysis and visualization purposes. Since Stripenn detects stripes based on principles of the image processing field, the first step is to convert each chromosome’s contact matrix, which is provided in the cooler file format^28^, to a digital image. Next, contrast adjustment and noise reduction are applied to a sliding window of 400 pixels along a diagonal line of the image (Fig. 1a). To enhance the contrast between signal and background, brightness is adjusted for multiple levels. To reduce noise, Gaussian blur effect can be applied to each brightened image. Then, Canny edge detection algorithm^24^, which is the most popular edge detection technique to date, is applied to processed images. The Canny edge detection algorithm, which is the backbone of Stripenn, relies on computing the gradient intensity of an image and applying various gradient magnitude thresholding to minimize spurious response to edge detection (see “Methods”). An edge in any digital image can point to a variety of directions; hence, the Canny algorithm uses filters to detect horizontal, vertical, and diagonal edges. Considering that contact matrices are symmetrical about the diagonal line, Stripenn only reports vertical edges predicted by the edge detection algorithm. Since a stripe is defined by a genomic anchor positioned on the diagonal line, predicted vertical edges longer than 10 pixels are connected to the diagonal line. Canny edge detection predicts two edges for each stripe and adjacent lines within a pre-defined distance are paired, forming a single vertical stripe. Together, Stripenn adapts principles from digital image processing and edge detection techniques to demarcate architectural stripes.

### Two scoring systems: *P*-value and stripiness

To quantitatively evaluate an architectural stripe, we devised two scoring systems: median *P*-value of pixel contrast and stripiness (Fig. 1a, b). The median *P*-value is the median of *P*-values of rows in a stripe and is used to evaluate the contrast between a predicted stripe and its neighbors (Fig. 1a and Supplementary Fig. 1a). Each *P*-value represents the significance of the pixel differences between a given row and its left or right neighboring pixels. To reduce the noise effect, we applied data smoothing by calculating the mean intensity of interactions in a window of 50 kb height around a given stripe row. Moreover, the mean intensity of left (*L*) and right (*R*) neighboring pixels are calculated from 50 kbp × 50 kbp windows adjacent to a given row. To estimate the *P*-value of the difference between the mean intensity of neighboring windows from the center ($C-L$ and $C-R$), a null distribution of pixel difference is created for 1000 random genomic regions. Since the expected contact frequency decreases for long-range interactions, null distributions are separately created from one to 400 pixels away from the diagonal line. To avoid overestimating the median *P*-value, the least significant value is chosen among left and right *P*-values. Hence, the median *P*-value is a metric of contrast and is used to filter low-quality stripes.

Although *P*-value calculation examines the contrast between a predicted stripe and its neighboring pixels, this metric is not capable of assessing the continuity of interactions within such structures. To penalize discontinuous stripes, which may represent loops, we devised another metric referred to as stripiness (Fig. 1b). Stripiness relies on subtraction of the gradients in vertical and horizontal directions and considers the contrast between a stripe and its neighbors, pixel continuity within a stripe, as well as a stripe’s median pixel value (see Methods). Supplementary Fig. 1b illustrates examples of stripes with various stripiness. Together, our algorithm exploits *P*-value and stripiness as scoring criteria to quantitate intensity, continuity, and contrast of predicted architectural stripes.

The principles of the Canny edge detection algorithm engineered in Stripenn can be easily applied to chromatin conformation measurements of different technologies including Hi-C^4^, HiChIP^25^, and Micro-C^26^ (Supplementary Fig. 2a). A larger number of stripes was predicted from Micro-C compared with Hi-C and HiChIP, suggesting that an increased resolution view of 3D genome structure can enable us to investigate more detailed features relevant to gene regulation. The larger number of predicted stripes from this assay is consistent with the notion that Micro-C can overcome the current resolution gap of Hi-C at the fine scale^26^. In addition, the application of Stripenn to Hi-C data in Drosophila^29^ yielded 137 architectural stripes, suggesting the utility of our method in non-mammalian genomes (Supplementary Fig. 2b).

### Benchmarking of Stripenn, Zebra, Chromosight, and domainClassifyR

We next aimed to systematically compare Stripenn with existing methods including Zebra^13^, Chromosight^21^, and domainClassifyR^12^. Since Zebra’s algorithm has a Github implementation developed by an independent group, which is called StripeCaller^22^, we first compared predictions of StripeCaller with those provided in the original study which proposed Zebra^13^. Although StripeCaller predicted a large number of stripes (6458) (Supplementary Fig. 2c), visualizing genomic interactions at predicted regions unique to StripeCaller did not corroborate the formation of strong stripes (Supplementary Fig. 2d, e). Hence, in the systematic comparison of Stripenn with existing methods, we used predictions provided in the original study reporting Zebra using Hi-C data in activated B cells^13^. In this comprehensive comparison, we found that 321 stripes were detected by domainClassifyR, 1757 by Stripenn, 4734 by Zebra, and 10,875 by Chromosight. This analysis also revealed that 241 genomic regions were predicted to form architectural stripes consistently across all methods. Representative examples of predicted stripes portrayed on Hi-C contact maps were indicators of sensitivity and specificity of these techniques (Fig. 1c). Prototypical stripes at TAD boundaries, such as the one harboring *Bcl6*, were detected by the four methods (Fig. 1c white stripe in the left panel). However, intra-TAD stripes, which consist of more than 70% of all stripes (Supplementary Fig. 2f) and mostly have positive stripiness values (Supplementary Fig. 2g), could not be detected by domainClassifyR. Representative examples of top-ranked domainClassifyR-specific stripes did not demonstrate strong continuous interactions at these predicted loci (Fig. 1c, gray stripe in the right panel). Although Zebra and Chromosight appeared to be sensitive techniques predicting larger number of stripes compared with other methods, these algorithms sacrificed specificity since genomic regions with weak stripy features such as loops or corner dots were occasionally predicted as stripes (Fig. 1c, yellow stripe in the second panel for Zebra and sky blue dots in the right panel for chromosight).

To complement the visual inspection of representative stripes, we next systematically compared the average intensity of interactions across stripes predicted by the four tools using pileup plots. This comparison showed that most domainClassifyR predictions did not form stripy features compared to predictions from other methods (Fig. 1d). Moreover, Chromosight and Zebra pileup plots demonstrated weak stripy patterns. In contrast, stripes predicted by Stripenn had strong intensity and significantly higher contrast represented by the lower level of contacts around genomic regions adjacent to predicted genomic coordinates. Together, the analysis of average genomic interactions suggests that Stripenn outperforms existing stripe callers.

To evaluate the quality of individual stripes across different methods rather than their average behavior, we next used the stripiness metric. We were not able to include domainClassifyR and Chromosight in this comparison since neither method provides information on the exact genomic coordinates of stripe domains: While domainClassifyR reports TAD coordinates, Chromosight provides a point on a stripe. Hence, we compared Stripenn and Zebra in terms of stripiness and stripe intensities. First, we collated the top 50 to 500 stripes based on the highest average stripe intensity (observed over expected) predicted by Stripenn and Zebra. In all cases, predicted stripes by Stripenn had significantly higher stripiness than predicted stripes by Zebra, suggesting higher quality of predictions by Stripenn compared with those of Zebra (Supplementary Fig. 2h). Next, we directly calculated the stripiness of common and unique predictions from Stripenn and Zebra. This comparison revealed that while the consensus predictions between Zebra and Stripenn in addition to Stripenn-specific stripes showed positive stripiness (Supplementary Fig. 2i), the average stripiness of Zebra-specific stripes was negative, indicating their low quality (Fig. 1e). In addition, Zebra-specific stripes harbored significantly lower interactions than those of Stripenn, which resulted in weaker stripes in the pileup plot analysis (Fig. 1d, f, S2j). Of note, all four methods are computationally efficient although the high-quality stripe detection of Stripenn takes longer and uses larger memory compared with other methods (Supplementary Fig. 3a–c). Since Stripenn perceives contact maps as digital images, the performance of stripe detection depends on sequencing coverage and hence the contact matrix resolution. From the downsampling analysis of Hi-C data in activated B cells, which includes more than 200 million valid pairs^13^, we observed that high-quality stripes could be predicted with at least 100 million valid pairs (Supplementary Fig. 3d, e). In summary, our comprehensive benchmarking of Stripenn demonstrates higher performance of Stripenn compared with existing stripe callers.

### Architectural stripes are favored on transcriptionally active regions

**Fig. 2:**
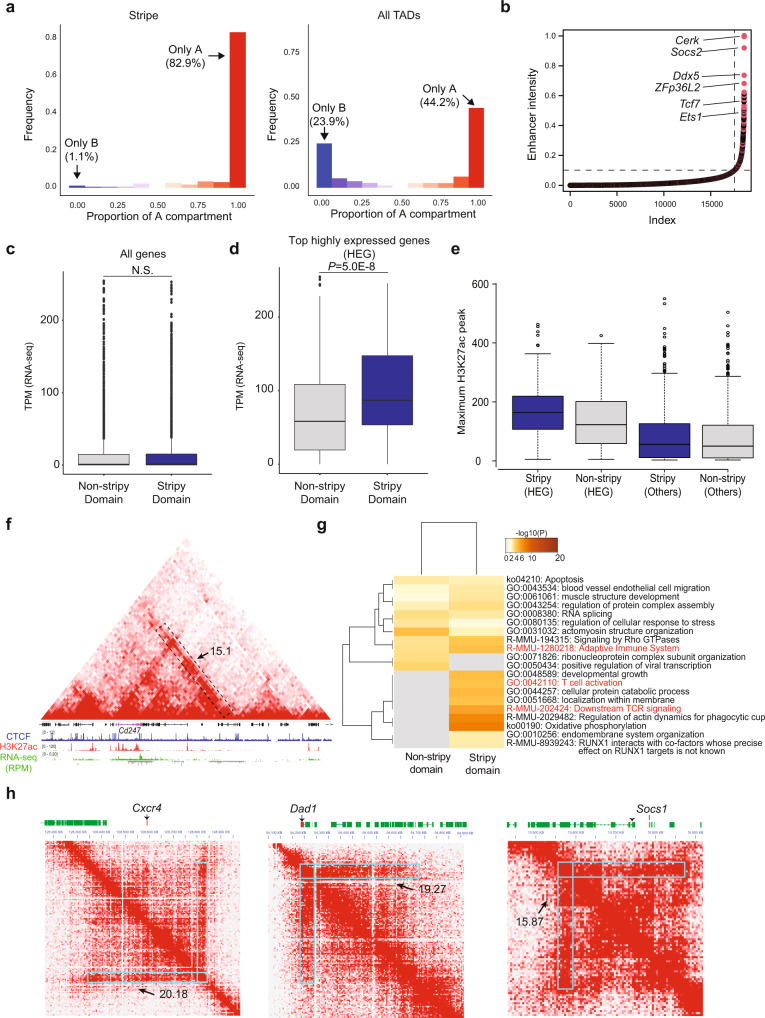
Stripes are located on transcriptionally active regions. **a** Genomic fraction of stripe domain overlapping with A compartment in activated B cell Hi-C data. The majority of stripes (82.9%) are located on the A compartment (left), unlike TADs which are distributed evenly between two compartments (right). **b** For DP thymocytes, the enhancer intensities in genomic bins (12.5 kb) are sorted. Typical (lower left) and superenhancers (upper right) are demarcated by dashed lines. Genomic bins overlapping with stripes are marked in pink. Some of the most highly expressed genes in each bin are shown. **c** The comparison of all gene expressions in stripy (blue) and non-stripy (gray) TADs. Data are shown as boxplots (centre, median; box limits, upper (75th) and lower (25th) percentiles; whiskers, 1.5× interquartile range; points, outliers). Statistical significance was tested using two-sided Wilcoxon rank sum test. **d** The comparison of the most highly expressed gene expression levels between stripy (blue) and non-stripy (gray) TADs. Data are shown as boxplots of which format is identical to (**c**). Statistical significance was tested using two-sided Wilcoxon rank sum test. **e** The distribution of the maximum H3K27ac peak within the promoter (±1 Kb of TSS) of most highly expressed genes (HEG) and others in stripy and non-stripy TADs. Data are shown as boxplots of which format is identical to (**c**). **f** An example showing active enhancer mark H3K27ac is biased to a highly expressed gene (*Cd247*). Stripe is marked with a dashed line, and stripiness (15.1) is shown. In the RNA-seq track, green and gray tracks represent the RPM on the first and second strands, respectively. **g** The gene ontology analysis of the most highly expressed genes in stripy and non-stripy TADs using Metascape. Immune response-related terms are marked red. **h** Examples of Thymocyte-specific genes (*Cxcr4, Dad1*, and *Socs1*) included in stripes from DP thymocyte. Srtripiness are shown for corresponding stripes.

The relevance of architectural stripes to gene expression has been examined in studies that utilized Zebra^13^ or domainClassifyR^12^ as stripe callers. To evaluate Stripenn’s performance across different technologies, we first investigated Stripenn’s predictions on Hi-C measurements from activated B cells. Using median *P*-value < 0.05, stripiness > 0, and 5 kb-resolution, we found 537 stripes in activated B cells (390 5′- and 147 3′-stripes). We examined the compartmentalization of stripes and found that stripes were frequently (~83%) formed on the transcriptionally active A compartment, which is consistent with the previous report using Micro-C measurements relying on stripy features at TAD boundaries^26^ (Fig. 2a). Only 1.1% of the stripes were detected in the B compartment and around 16% of stripes spanned A and B compartments (Fig. 2a). This selective enrichment of stripes on A compartment contrasts with the compartmentalization of TADs where only half of TADs (44.2%) are positioned in the A compartment, emphasizing the association of stripes with the active chromatin state (Fig. 2a). These findings are consistent with the observation that stripes frequently accommodate super-enhancers^12,13^. We further focused on TADs harboring stripes, referred to as “stripy” TADs, and compared them with those without any stripes, referred to as “non-stripy” TADs. We examined genomic length and the enrichment of architectural proteins including CTCF, cohesin subunits SMC3, RAD21, and cohesin loader NIPBL in the two TAD classes. The stripy TADs were defined as TADs containing any stripe with median *P*-value < 0.05 and stripiness >0. The non-stripy TADs did not include any stripes predicted by Stripenn and only TADs on the A compartment were considered in this comparison. Together, 155 stripy and 286 non-stripy TADs were considered for further analysis. Interestingly, we found that stripy TADs were significantly longer in genomic length compared with non-stripy TADs (Supplementary Fig. 4a). TADs in the A compartment were on average smaller in genomic length compared with those on the B compartment (Supplementary Fig. 4c, d) and some stripes spanned both A and B compartments (Fig. 2a). Hence, we compared the genomic length of stripy and non-stripy TADs in the A compartment and found that the disproportionate difference in genomic length was also present when stripy TADs exclusively in the A compartment were considered for this comparison (Supplementary Fig. 4b). Together, the link between genomic length and stripe formation on the active compartment may imply the accommodation of numerous regulatory elements in stripy TADs. We next compared the median intensity profiles of architectural proteins on stripy and non-stripy TADs and found that architectural proteins such as CTCF, SMC3, and, RAD21 had higher occupancy on stripy TADs compared with non-stripy TADs (Supplementary Fig. 4e, f). Consistent with previous studies^12,13^, CTCF and cohesin subunits were highly enriched at stripe anchors (Supplementary Fig. 4g). Moreover, depletion of the cohesin subunit (Rad21) led to a significant reduction of stripes (2255 in wild type and 118 in Rad21-depleted cells) in HCT116 cell lines (Supplementary Fig. 4h, i). Similarly, mutation in CTCF protein resulted in a slightly reduced stripes (941 in wild type and 722 in mutant cells). This moderate effect on stripes can be linked to a significant number of CTCF binding events, which were still bound on the genome in cells expressing CTCF mutants^30^. These data suggest that stripes favor the transcriptionally active A compartment and the occupancy of architectural proteins at stripy TADs may implicate their roles in stripe formation.

### Stripy TADs are more accessible and possess more active enhancers than non-stripy TADs

To further assess the quality of architectural stripes detected by chromosome conformation techniques other than Hi-C (Supplementary Fig. 2a), we applied Stripenn to HiChIP measurements. HiChIP is a ligation-proximity reaction assay, which detects interacting DNA fragments bound by a protein of interest^25^. We applied Stripenn to our recently generated SMC1 HiChIP in double positive (DP) thymocytes in C57BL/6J mice^31^ and detected 431 stripes (314 5′- and 117 3′-stripes) (median *P*-value < 0.05 and stripiness >0). Consistent with findings based on Hi-C maps of B cells, we confirmed that most stripes formed on the A compartment of DP thymocytes (Supplementary Fig. 5a). Moreover, we found that deposition of active enhancer mark, H3K27ac, was skewed towards stripe anchors (Supplementary Fig. 5b) and the active enhancer marks were significantly more enriched at stripy TADs compared to non-stripy TADs (Supplementary Fig. 5c). In agreement, stripy TADs were on average more accessible than non-stripy TADs (Supplementary Fig. 5d). Considering the enrichment of active enhancer signature within stripes, we ranked the genomic bins based on the enhancer intensity and classified them as 1013 super-enhancers and 17,555 typical-enhancers. Consistent with previous findings, stripes had significant overlap with super-enhancer regions (300/1013 bins; hypergeometric test *P*-value = 0, Fig. 2b). Interestingly, genes harboring architectural stripes and involved in T-cell biology were highly ranked in terms of H3K27ac deposition and enhancer intensity. For example, the gene which was ranked third, *Socs2*, regulates the T helper cell type 2 (Th2) and the pathogenesis of type 2 immune responses such as asthma. Another highly ranked gene, *Ddx5*, is required for the cytokine production of T helper cell type 17 (Th17). Moreover, *Tcf7* a transcription factor for early T cell development setting up the chromatin accessibility landscape of T cells encompassed an architectural stripe^32^. Together, these data further corroborate that architectural stripes favor highly accessible and active chromatin states at genes with prominent roles in cell-type-specific processes.

### Stripy TADs are formed at highly expressed genes associated with T cells

To assess the association between stripe formation and transcriptional outputs, we compared the expression levels of genes located on 156 stripy TADs versus 144 non-stripy TADs within the A compartment of DP thymocytes. On average, we did not find any significant difference between the expression levels of genes located on stripy vs. non-stripy TADs (Fig. 2c). Nonetheless, we found that the most highly expressed genes encompassing stripy TADs were significantly more expressed than the most highly expressed genes encompassing non-stripy TADs (Fig. 2d). This finding suggests that not all but distinct genes in stripy TADs benefit from the continuous interaction of a stripe anchor with regulatory elements located on a genomic domain. To further study this phenomenon, we assessed the distribution of active enhancer marks in stripy and non-stripy TADs. We observed that only at highly expressed genes, the H3K27ac recruitment was significantly higher in stripy TADs compared with non-stripy TADs (Fig. 2e). An example includes a genomic locus accomodating 12 genes where only one gene, *Cd247*, is highly expressed, harboring an intense level of H3K27ac (Fig. 2f). Since *Cd247* has a salient function as a surface protein in T cells, we interrogated if other highly expressed genes associated with stripes also have functional relevance in T cells. To delineate the identity of these genes, we performed gene ontology analysis using Metascape^33^. The most highly expressed genes in either stripy or non-stripy TADs were enriched in terms such as ‘apoptosis’^34^, ‘actomyosin structure organization’^35^, ‘Signaling by Rho GTPases’^36^. Interestingly, genes located on stripy TADs were more significantly enriched in the adaptive immune response-related terms such as ‘T cell activation’ (*Cd3g, Malt1, Myh9*, and *Thy1*), ‘adaptive immune system, downstream TCR signaling’ (*Btrc, Cd247, Cd3g, Clec2d, Cul3, Dync1li1, Malt1, Psma4, Psmb8, Psme3, Tab2*, and *Ubr4*), and ‘oxidative phosphorylation’^37^ (*Atp6v0a2, Cox7a2l, Ndufa9*), compared with genes on non-stripy TADs (Fig. 2g). This analysis indicates that genes important in T cell signaling and activation are frequently accompanied with architectural stripes. Representative examples demonstrate the interactions of stripe anchors and T cell-associated genes such as *Cxcr4*, *Dad1*, and *Socs1* (Fig. 2h). The gene encoding the chemokine receptor, CXCR4, which is recruited into immune synapse during T cell activation^38^, is in the middle of a stripy TAD and can act as a boundary of two nested TADs. Another representative architectural stripe is located on an anti-apoptosis gene *Dad1*, which can enhance T-cell proliferation^39^, and is located downstream of the T-cell receptor alpha chain gene (TCRα)^40,41^. Similar to *Cxcr4*, *Socs1* required to suppress the cytokine signaling, regulating T cell proliferation, activation, and function^42^, resides in the middle of a stripy TAD and is preferentially located near the boundary of two nested TADs. The selective enrichment of T cell-associated genes within architectural stripes of T cells further confirms the potential cell-type-specific regulatory role of stripes.

### Stripes are strongly conserved between two inbred mouse strains

**Fig. 3:**
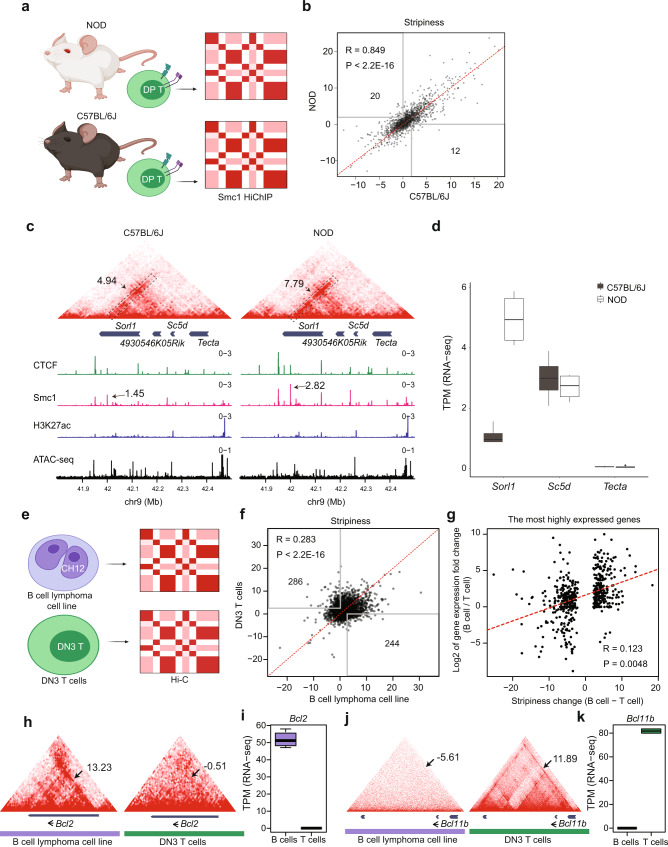
Stripes are mostly preserved between T-cells of two inbred mouse strains but highly different between B and T cells. **a** Stripes were extracted from SMC1-HiChIP data of DP thymocyte of control (C57BL/6J) and prediabetic (NOD) mice. **b** Stripenn predictions in two strains were merged, and then stripiness of all stripes were recalculated based on C57BL/6J and NOD HiChIP data. The number of NOD and C57BL/6J-specific stripes (stripiness > 2 in one and <0 in the other condition) was 20 and 12, respectively. Peaerson correlation coefficient (R) and corresponding *P*-value (*P*) are shown (two-sided and multiple hypothesis tesing was not applied). **c** An example of a conserved stripe across two strains, including a differentially expressed gene (*Sorl1*). Stripiness of two stripes are represented in the heatmap. A representative differential SMC1 peaks between C57BL/6J and NOD is marked as well. **d** The expression of genes within the TAD in (**c**) are represented for both C57BL/6J (*N* = 4 biological replicates) and NOD (*N* = 4 biological replicates). Data are shown as boxplots (centre, median; box limits, upper (75th) and lower (25th) percentiles; whiskers, maximum and minimum). **e** Stripes were extracted from the Hi-C data in CH12 B lymphoma cell lines and newly generated Hi-C data in DN3 T cells. **f** Stripiness was measured for union stripes from B and T cells. The number of B and T cell-specific stripes are 244 and 286, respectively. Two-sided Pearson correlation test was performed and multiple hypothesis testing was not applied. **g** The correlation between the gene expression fold change (B cells/T cells) and stripiness change (Stripiness in B cells–Stripiness in T cells) was estimated for the B and T cell-specific stripes. Here, the most highly expressed genes in each stripe were used to calculate each stripe’s gene expression fold change. Two-sided Pearson correlation test was performed and multiple hypothesis testing was not applied. **h** An example for a B cell-specific stripe at the *Bcl2* locus. Arrows point to the *Bcl2* stripe position, and stripiness assessing the strength of this stripe in (**b**) T and B cells is provided. **i** The expression of *Bcl2* is significantly higher in B cells (*N* = 4) than T cells (*N* = 2). Data are shown as boxplots (centre, median; box limits, upper (75th) and lower (25th) percentiles; whiskers, maximum and minimum). **j** An example T cell-specific stripe containing *Bcl11b*. Arrows point to the *Bcl11b* stripe positions. **k** *Bcl11b* expression is significantly higher in T cells (*N* = 2) than B cells (*N* = 4). Data are shown as boxplots (centre, median; box limits, upper (75th) and lower (25th) percentiles; whiskers, maximum and minimum).

We next aimed to investigate whether genetic variation can alter stripe formation and be linked to transcriptional regulation. Hence, we relied on millions of natural genetic variation between two inbred mouse strains, the C57BL/6J and nonobese diabetes (NOD) mice. Using SMC1 HiChIP measurements in DP thymocytes, Stripenn was able to detect 953 and 1151 stripes in C57BL/6J and NOD mice, respectively^31,43^ (Fig. 3a). To compare the degree of interactions between two strains, stripiness, and median *P*-values of stripes merged across two strains were calculated. Despite more than 5 million single-nucleotide polymorphisms and 400 thousand insertions and deletions, the stripiness of predictions in DP thymocytes of two mouse strains was significantly correlated (Fig. 3b and Supplementary Fig. S6a). The visual inspection of loci encompassing major T cell-associated genes such as *Bcl6* and *Ets1* demonstrated a large-scale conservation of architectural stripes between two strains in DP T thymocytes (Supplementary Fig. 6b-c).

Despite this global similarity of stripes between two strains, the stripiness metric demonstrated exceptional stripes with enhanced interactions in a strain-specific manner. One example is a stripe specific to NOD strain harboring killer cell lectin-like receptor (*Klr*) gene family located on chromosome 6 (Supplementary Fig. 6d). These genes, which are typically expressed in natural killer cells, identify and enable the destruction of virus-infected cells^44^. Our own group recently showed that this cluster of genes forms a set of long-range genomic interactions among enhancers and promoters, also referred to as hyperconnected 3D cliques^31^. These genes were lowly expressed in T cells of C57BL/6J but showed consistently higher expression in NOD mice (Supplementary Fig. 6e). In addition, the intensity of CTCF, SMC1, and H3K27ac as well as DNA accessibility were much higher in the NOD strain (Supplementary Fig. 6d). Another NOD-specific stripe contained genes such as *Aim2*, *Ifi203*, *Ifi204, Ifi205*, *Mndal*, and *Olfr421-ps1* on chromosome 1 (Supplementary Fig. 6f). Protein products of *Aim2* are involved in inflammasomes and act as sensors for pathogen or abnormal DNA in the cytoplasm^45^. An NOD-restricted stripe crossing *Aim2* was predicted by Stripenn and the visual inspection of this locus confirms significantly enhanced interactions in NOD compared with C57BL/6J. Moreover, CTCF, SMC1, and H3K27ac modification were significantly more abundant at the anchor of the NOD-specific stripe only in NOD mice. Consistent with enhanced chromatin interactions at this locus, all genes except *Mndal* in this stripy region showed higher expression levels in NOD compared with C57BL/6J (Supplementary Fig. 6g).

### Subtle changes in local interactions are linked to differential gene expression

Since the majority of stripes were conserved between two strains, we next examined the relevance of stripe formation at genomic regions harboring differentially expressed genes between two strains. The differential gene expression analysis yielded 664 and 633 genes upregulated in NOD and C57BL/6J, respectively (DESeq2 adjusted *P* value < 0.05, log2 fold change > 1). Nonetheless, we were not able to detect a significant difference in interactions of stripy regions between the two strains accommodating differentially expressed genes (Supplementary Fig. 6h). A representative example at the *Sorl1* locus demonstrates an exception to this trend. *Sorl1* is highly expressed in thymocytes of NOD compared with C57BL/6 J mice (adjust *P* value = 2.09e−17). Although the architectural stripe formed at this locus was detected by Stripenn in both strains, the stripiness of this stripe was ~2 times higher in NOD compared with C57BL/6J (Fig. 3c; highlighted region). The stronger enrichment of SMC1 might have increased enhancer and promoter interactions in this region, leading to higher expression levels of *Sorl1* in NOD mice^46^. This example further suggests that subtle changes in local chromatin interactions, which are detectable by Stripenn, may be linked to changes in gene expression. Overall, architectural stripes are highly conserved features of a given cell type.

### Cell-type-specific stripes from B and T cells are related to differential gene expression

The finding that architectural stripes are mostly conserved in T cells of two different mouse strains implies that stripes form at genes closely related to cell identity, which are largely resilient to natural sequence variation. We next compared the association of stripes and gene expression between two developmentally related but distinct cell types, namely B and T lymphocytes. We used publicly available Hi-C measurements in a B cell lymphoma cell line^13^ (CH12) and performed Hi-C experiments in a T cell line representing T cell progenitors (DN3) (Fig. 3e). Stripenn was able to detect 1787 and 1580 stripes in B and T lymphocytes, respectively (median P-value < 0.05). Unlike the comparison of T cells between the two strains of mice, we found significant differences in stripe formation between T and B lymphocytes (Fig. 3f–k). The stripiness (and median *P*-value) of predicted stripes between T and B lymphocytes showed low correlation (*R* = 0.283 for stripiness and *R* = 0.126 for median *P*-value; Fig. 3f and Supplementary Fig. S7a). Accordingly, the number of cell-type-specific stripes between B and T cells was significantly higher than those of strain-specific stripes (B cell-specific: 244, T cell-specific: 286, C57BL/6J specific: 12, NOD specific: 20; Fig. 3b, f). We next compared differences in stripiness between B and T cells and differences in gene expression levels between B and T cells. Using the expression levels of the most highly expressed gene in each stripe or the average expression levels across stripes, we found a significant correlation between differences in stripiness and differences in gene expression between T and B cells (Fig. 3f and Supplementary Fig. S7b, c). The comparison of differences in the total sum of observed over expected interaction levels within stripes and differences in gene expression levels also demonstrated significantly high correlation between two cell types (Supplementary Fig. 7d–f).

Genomic regions harboring *Bcl2* and *Bcl11b* genes were two representative stripes that demonstrated strong cell-type-specific stripe formation and transcription in B and T cells, respectively (Fig. 3h–k). The *Bcl2* gene is exclusively expressed in B cell lymphoma cell line and associates with a stripe that spans the entire gene body (Fig. 3h, i). *Bcl2*, a key regulator of apoptosis, is required for lymphoma development and the alteration in *Bcl2* family is widely observed in B cell lymphoma^47^. In contrast, *Bcl11b* is selectively expressed in T cells and a T cell-specific stripe is formed at the genomic locus harboring this gene (Fig. 3j). BCL11B is an essential checkpoint protein during multiple steps in the T cell development^48^. Moreover, gene ontology analysis showed the most highly expressed genes in B and T cell-specific stripes were selectively enriched in terms related to the biology of B and T cells (Supplementary Fig. 7g, h). Collectively, these data indicate that the cell-type-specific formation of stripes is closely related to the cell-type-specific control of gene expression.

### Evolutionarily conserved stripes form at cell-type-specific genes

**Fig. 4:**
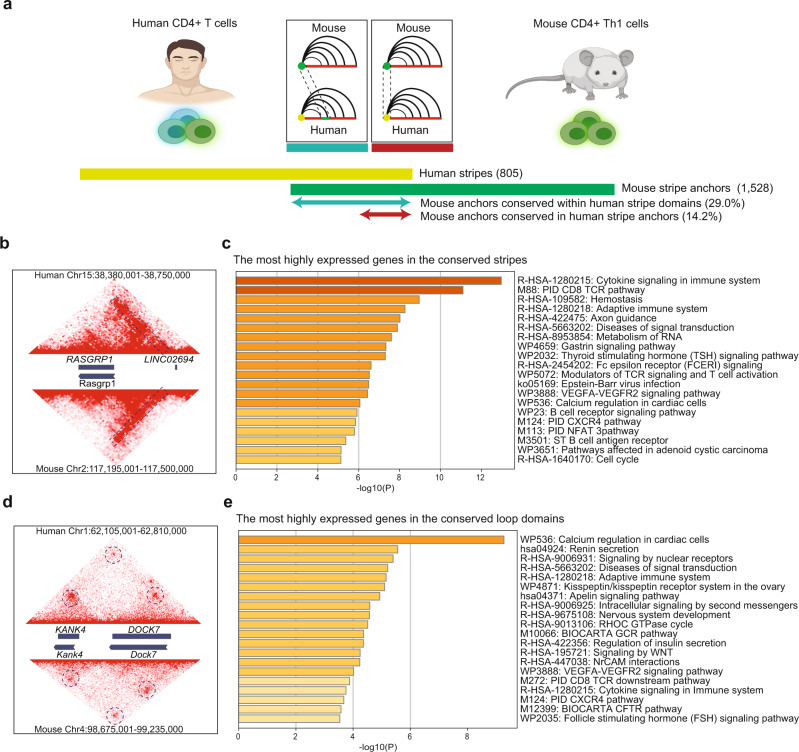
Conserved stripes between human and mouse are cell-type-specific. **a** The conservation of stripes in human CD4^+^ T cells and mouse CD4^+^ Th1 cells is examined. For human and mouse data, 805 and 1528 stripes were detected, respectively. When mouse stripe anchors (mm10) were converted to human orthologous coordinates (hg38) and compared to human stripe anchors, 217 (14.2%) were conserved. Similarly, 443 (29.0%) of mouse stripe anchors were conserved within human stripe domains. **b** An example stripe conserved across human and mouse harboring *RASFRP1*. **c** Gene ontology analysis of the most highly expressed genes in the conserved stripes was performed using Metascape which utilizes hypergeometric test and Benjamini–Hochberg *P*-value adjustment. **d** An example loop conservation across human and mouse at *KANK4-DOCK7* loci. **e** Gene ontology analysis of the most highly expressed genes in the conserved loop domains was performed using Metascape.

To further evaluate the conservation of stripes across species, we compared stripe formation between humans and mice in CD4^+^ T cells. Naive CD4^+^ T cells, which are critical components of the adaptive immune system, can differentiate into various subsets such as T helper 1 (Th1) and Th2, facilitating the elimination of distinct pathogens^49^. We generated ultra-deep Hi-C maps in CD4^+^ Th1 cells in mice and CD4^+^ T cells in humans. Stripenn was able to detect 1528 stripes in mouse CD4^+^ T cells while 805 stripes were detected in human CD4^+^ T cells. First, we defined the stripe anchors in mouse CD4^+^ T cells that had orthologous regions in the human genome (1414, 92,5% of mouse stripes). We found that 14.2% of mouse stripe anchors were conserved as human stripe anchors while 29.0% were conserved within human stripe domains (Fig. 4a). For example, a stripe containing *Rasgrp1*, a T cell-specific nucleotide exchange factor activating the MAPK pathway^50^, is conserved across humans and mice (Fig. 4b). To compare stripes and loops from the perspective of conservation between humans and mice, we detected loops from mouse and human CD4^+^ T cell using Mustache^51^. This analysis suggested 2040 loops in humans while 6498 loops were detected in mice CD4^+^ T cells (FDR < 0.01). Among the 2041 loops detected in mouse T cells, which were not predicted as stripes, 398 loops were conserved as loops in human T cells, showing similar a conservation level (~22%, Fig. 4d). Next, we annotated the genes within conserved stripes and loops. Of note, the stripes and loops unique in each species were not considered for further analysis since the source of divergence could be related to differences in cell states. Interestingly, the most highly expressed genes in the conserved stripe domains were strongly enriched in terms related to T cell biology such as ‘Cytokine signaling in immune system’ and ‘T cell receptor pathway’ and ‘Adaptive immune system’ (Fig. 4c). In contrast, the most highly expressed genes in the conserved loop domains were weakly associated with terms related to T cell biology (Fig. 4e). Together, the comparison of stripes in CD4^+^ T cells in humans and mice suggests an evolutionarily conserved pattern where genes that determine cell identity are enriched at architectural stripes.

## Discussion

Since its invention in 2009^4^, Hi-C and its variations suffered from the unreasonably high cost of sequencing required to map high-resolution features such as enhancer-promoter interactions. With the cost of sequencing dropping significantly due to sequencing platforms such as NovaSeq and the emergence of single-nucleosome resolution techniques such as Micro-C^26^, the 4D nucleome community has witnessed the emergence of fine structures such as architectural stripes, which are not detected in low-resolution 3D genome measurements. Despite the pioneering work which suggested that cohesin continually extrudes loops of chromatin in vivo to form architectural stripes relying on ATP to fuel loop extrusion^13^, detailed molecular processes through which some regions form stripes, but not stable loops, remain unclear. A major technical barrier to study these features and compare them across various developmental programs is the lack of a computational method to accurately demarcate and quantitate architectural stripes. Here, reasoning that stripes resemble edges of a digital image, we developed a technique with high accuracy and sensitivity relying on the most popular and widely-used edge detection algorithm in computer vision. The comparison of this technique with previously developed stripe callers revealed that relying on edge detection algorithms enables the identification of accurate architectural features. Consistent with previously published studies, we demonstrated that architectural stripes are enriched at transcriptionally active and accessible genomic regions. Moreover, we mapped these features in inbred strains of mice and found the large-scale conservation of architectural stripes despite millions of single nucleotide variation, suggesting the resilience of architectural stripes to sequence variation. We further corroborated that stripes are preferentially formed at lineage-determining genes through the comparison of B and T cells, as well as the stripe conservation analysis across humans and mice.

A critical yet unanswered question relates to mechanistically distinguishing stripes from chromatin loops. It remains unclear how architectural stripes selectively form in a particular cell type but can represent as corner dots or chromatin loops in other cell types. It remains unlikely that clustering of CTCF recognition sites and their orientation can play a role considering the identical underlying DNA sequence in different cell types. Whether cell-type-specific CTCF binding events dictated by cell-type-specific accessible chromatin regions at stripe anchors or additional epigenetic modifications such as DNA methylation can earmark the formation of stripes in select cell types remains to be shown. Exploiting machine-learning strategies and using large-scale detection of architectural stripes by Stripenn across different cell types collected by the 4D nucleome community can pave the way to better understand the grammar of stripe formation. The utilization of live cell imaging and chromatin tracing^52^ can further allow us better define the dynamic of stripe formation in time and space.

## Methods

### Experimental model and subject details

Our research complies with all relevant ethical regulations based on University of Pennsylvania’s guidelines.

### Cells and culture conditions

Scid.adh cell line, a pro-T (DN3 stage) cell line derived from spontaneous thymic lymphomas (Carleton et al. ^53^), was a kind gift from Warren Pear’s lab. These cells were grown in RPMI 1640 medium (Invitrogen), supplemented with 10% fetal bovine serum (FisherScientific), 1 mM sodium pyruvate (Gibco), 1% non-essential amino acids (Gibco), 2 mM L-Glutamine (Lonza), 1% penicillin–streptomycin and 0.1% 2-Mercaptoethanol (Gibco). All cells were grown at 37 °C and 5% CO_2_.

Total cells were isolated from mouse spleen and lymph nodes and naive CD4+ T cells were enriched using negative selection beads (STEMCELL, Cat# 19765). These cells were cultured in RPMI 1640 medium (Invitrogen), supplemented with 10% fetal bovine serum (FisherScientific), 1 mM sodium pyruvate (Gibco), 1% non-essential amino acids (Gibco), 1X GlutaMAX (Gibco), 1% HEPES (Gibco), 1% penicillin–streptomycin and 0.1% 2-Mercaptoethanol (Gibco) and in-vitro polarized to Th1 cells by treating with 1ug/ml anti-CD3 (BD Biosciences, cat#553294), 1ug/ml anti-CD28 (BD Biosciences, cat#553294), 10 ng/ml recombinant IL-12, 1 ng/ml recombinant IL-2 for 6 days. All animal work was in accordance with the Institutional Animal Care and Use Committee of the University of Pennsylvania in accordance with NIH guidelines. Human tissues were procured by the HPAP consortium (RRID:SCR_016202; https://hpap.pmacs.upenn.edu), part of the Human Islet Research Network (https://hirnetwork.org/), with approval from the University of Florida Institutional Review Board (IRB # 201600029) and the United Network for Organ Sharing (UNOS). All the specimens or data come from cadavers or otherwise deceased individuals, hence the University of Florida Institutional Review Board has determined that our study is not considered human subjects research and has therefore waived the need for informed consent and exempted the requirement to be conducted in accordance with the declaration of Helsinki. Human CD4^+^ T cells were sorted out lymph nodes of a healthy human donor collected by HPAP.

### Hi-C

Hi-C libraries were generated on 10^6^ cells using Arima-HiC^+^ kit (Arima Genomics) and Accel-NGS @S Plus DNA Library kit (21024 Swift Biosciences), according to the manufacturer’s recommendations. Libraries were validated for quality and size distribution using Qubit dsDNA HS Assay Kit (Invitrogen, cat# Q32851) and TapeStation 2200 (Agilent). Libraries were paired-end sequenced (66 bp+66 bp) on NovaSeq 6000 (Illumina).

Hi-C in Th1 cells: Total cells were isolated from mouse spleen and lymph nodes and naive CD4+ T cells were enriched using EasySep Mouse naive CD4+ T cell Isolation kit (STEMCELL, Cat# 19765). These cells were cultured in RPMI 1640 medium (Invitrogen), supplemented with 10% fetal bovine serum (HyClone), 1 mM sodium pyruvate (Gibco), 1% non-essential amino acids (Gibco), 1X GlutaMAX (Gibco), 1% HEPES (Gibco), 1% penicillin–streptomycin and 0.1% 2-mercaptoethanol (Gibco) and in-vitro polarized to Th1 cells by treating with 1 μg/ml anti-CD3 (BioLegend, cat#100301), 1 μg/ml anti-CD28 (BD Biosciences, cat#553294), 10 ng/ml recombinant IL-12 (BioLegend, cat# 577002), 1 ng/ml recombinant IL-2 (BioLegend, cat#575402) for 6 days.

Human CD4+ T cells were sorted out from pancreatic lymph nodes. Sorting was done after gating on Aqua- (live cells), CD14− CD19− (not B cells) CD3+ (T cells) CD4− CD4+ cells, using antibodies anti-human CD3 (BD biosciences, cat#565120), anti-human CD14 (BioLegend, cat#301842), anti-human CD19 (BioLegend, cat#302205), anti-human CD8 (BioLegend, cat#301040), anti-human CD4 (BioLegend, cat#317411).

### Computational analysis

#### Stripenn

Stripenn detects architectural stripes from chromatin conformation capture data using image processing principles and scores them with median *P*-value and stripiness. Stripenn can be installed via pip (or pip3) and more information on installation and usage is described on the GitHub page (https://github.com/VahediLab/stripenn).

*Input and output*: The input of Stripenn is in a *cooler* file format (.cool or.mcool) that contains a genomic matrix such as the Hi-C contact frequency matrix^28^. The output is a table of stripe coordinates, sizes, and measures (median *P*-value, stripiness, and average/sum of pixel values within stripes). Stripenn runs through three steps to generate the output: (1) to search for candidate stripes, (2) to measure median *P*-value, and (3) to measure stripiness.

*To search for candidate stripes***:** For each chromosome, stripes are searched within a series of windows of 400 pixels that are moving along a diagonal line of contact frequency heatmap using the step size of 200 pixels. In each window, referred to as submatrix hereinafter, rows and columns composed of only zeros are removed and the corresponding genomic regions are deleted. Stripenn follows eight steps to find candidate stripes in each submatrix:

1. *Convert matrix to image:* A submatrix is converted to an image, as shown in Fig. 1a. In this process, pixel values in a submatrix should be truncated to an appropriate value, which we refer to as *maximum pixel value*, to visualize the 3D chromatin structure properly. This step is important since extremely low or high maximum pixel value makes the image covered with only red or white pixels, which does not give any information. Because the stripe detection is sensitive to the maximum pixel value, Stripenn can search stripes for multiple maximum pixel values. These are determined by the percentiles of positive contact frequencies that the users provide as input. For example, the option ‘-m 0.93,0.95,0.97’ in the command line enables to set the maximum pixel values as top 7%, 5%, and 3% of the positive contact frequency values of each chromosome (default = ‘-m 0.95,0.96,0.97,0.98,0.99’). These percentiles should be set differently for different data based on the sequencing depth of the data. Stripenn’s ‘*seeimage*’ function helps the users decide the percentile values by visualizing a contact frequency matrix of given coordinates for a given percentile. Once maximum pixel value (*M*) is determined, each pixel value (*P*) in submatrix is then converted to RGB codes such as
  1 $$R=255{{{{{\rm{;}}}}}}\,G=B={\max }\left(\frac{255* \left(M-P\right)}{M},0\right)$$
  These RGB codes are then merged using the *merge* function in opencv-python package^54^ to create an image.
2. *Brightness adjustment*: Next, we adjust the brightness to increase the contrast between the signal and noise of the image. Because the optimal brightness that best reveals the 3D structure in the image is not known, we adjust the brightness for multiple levels and find stripes for each level. To do this, MATLAB’s *imadjust* function^55^ is implemented in python and used with changing *high_in* parameters from 0.5 to 1.0 increasing by 0.1.
3. *Blur effect*: Next, the mean filter can be applied to further reduce the noise of the image by changing the kernel size option (*bfilter*). Here, the *filter2D* function of the opencv-python package is used and a 3 × 3 two-dimensional matrix filled with 1/9 is used as a default kernel. The kernel size option can be set as 1 to turn off the blur effect.
4. *Canny edge detection*: The image is then converted to grayscale using the *cvtColor* function in the opencv-python package to apply Canny edge detection. It is the most widely used edge detection method because it searches optimal edges that satisfy three criteria of an optimal edge: (1) edge with a low error rate, (2) optimal localization, and (3) only one strong signal is detected. The edge image ***E*** is extracted using the *canny* function (default sigma = 2.0) in Scikit-image package^56^.
5. *Vertical line detection*: Because Hi-C data is symmetric about the diagonal line, horizontal stripes are automatically detected if corresponding vertical stripes are found. Thus, we search for only vertical stripes here. To do this, pixels whose orientations are not between 60° and 120° are removed. The orientation *θ* of each pixel ${{{{{\boldsymbol{E}}}}}}(i,j)$ is calculated using the Sobel operator as follows^57^.
  2 $${G}_{x}=\left[\begin{array}{ccc}-1 & 0 & 1\\ -2 & 0 & 2\\ -1 & 0 & 1\end{array}\right]\circ {E}_{k\times l},\,{G}_{y}=\left[\begin{array}{ccc}1 & 2 & 1\\ 0 & 0 & 0\\ -1 & -2 & -1\end{array}\right]\circ {E}_{k\times l}$$
  3 $$\theta ={\arctan }\left(\frac{{G}_{y}}{{G}_{x}}\right)$$
  Where ${G}_{x}$ and ${G}_{y}$ are gradient in *x*- and *y*-direction, and ${E}_{k\times l}$ is 3 × 3 submatrix of edge matrix ${{{{{\boldsymbol{E}}}}}}$ ($i-1\le k\le i+1$, $j-1\le l\le j+1$). Here, the *convolve2d* function in SciPy package^58^ and *arctan2* function in NumPy package^59^ are used. Because the orientations of edges in ${i}^{{th}}$ column are reflected in ${(i+1)}^{{th}}$ column of the orientation matrix, we shift the vertical line to 1 pixel left. Next, continuous pixels longer than 10 pixels are detected. This continuous pixel allows five gaps in maximum, and the length of continuous pixels can be modified by the option ‘*minL*’. Since the edges are usually not straight in reality, we allow each pixel in a straight line to shift left or right by 1 pixel. These continuous lines are then extended to the diagonal line.
6. *Line refinement*: After step 5, several lines can be combined since we allow a 1-pixel shift. To find the representative line among them, we first converted pixel one (signal) in each line to zero (background) if it is originally zero in the edge image ${{{{{\boldsymbol{E}}}}}}$. Then, we find the representative column as the average column indexes weighted by their line lengths.
7. *Adjacent line pairing*: Next, we pair two vertical lines if their distance is less than *N* pixels (default: 8 pixels). If there are multiple lines within a narrow window, those lines are paired in a serial way. Then, each pair is evaluated with a median *P*-value to filter low-quality stripes. The maximum stripe width should change according to the data resolution. For example, we recommend using 8 pixels and 16 pixels as maximum widths for 10 kb and 5 kb, respectively.
8. *Merging stripes*: Searching stripes from overlapping submatrices with multiple brightness parameters results in several duplicated stripes. In the last step of candidate stripe search, these are merged into one as the stripes having the largest length/width value.

#### Median *P*-value

The median *P*-value is devised to filter candidate stripes based on a pixel contrast between stripes and their backgrounds. Simply, the median *P*-value is a median of a series of *P*-values evaluated from each stripe row. The *P*-value of each stripe row is estimated as described in Supplementary Fig. 1a. First, the mean contact frequency ($C$) of a given row is calculated. Here, data smoothing is applied to reduce the noise by deriving the mean contact frequency from the 2-D matrix of $N$ × *width* pixels, where $N$ is the number of pixels corresponding to 50 kb that depends on the resolution of the data. Next, we calculate the mean contact frequency of both left ($L$) and right ($R$) neighborhoods of the testing row. The mean frequency is calculated from the 2D matrix of *N* × *N* pixels right next to the testing row. Since the *P*-value estimates the significance of $C-L$ and $C-R$, the corresponding null distribution is constructed by randomly selecting 1000 data points. Considering that the bin distance affects the contact frequency, we select data points of identical bin distance as testing row pixel (first pixel for 5′-stripe and last pixel for 3′-stripe). The null distributions of left and right neighborhoods are constructed separately so that the significance of $C-L$ and $C-R$ are accurately measured. To avoid overestimating the median *P*-value, we choose the less significant value. Stripenn output reports a list of stripes of which *P*-value is less than 0.1 as a default.

#### Stripiness

Median *P*-value measures the significance of the contrast between the stripe and the background; however, this metric does not reflect the contact frequency and continuity of intensity within the stripe. To make up for these shortcomings of median *P*-value, stripiness has been devised. Stripiness (S) is estimated on the observed/expected contact frequency matrix, and it is defined as

4 $$S=M\frac{{\sum }_{i}\Delta {G}^{i}}{L}$$

where $L$ is the number of stripe rows, $i$ is the index of stripe row ($1\le i\le L$), $M$ is the median of mean frequencies of stripe rows, and $\triangle {G}^{i}$ is gradient change at $i$^th^ stripe row. The gradient change $\Delta {G}^{i}$ represents the difference between gradient in the *x*-direction (across stripe and background) and *y*-direction (within stripe). Since larger gradient in the *x*-direction and smaller gradient in the *y*-direction are features of strong stripes, we define $\Delta {G}^{i}$ as

5 $$\triangle {G}_{i}={\min }\left({G}_{x,L}^{i},\,{G}_{x,R}^{i}\right)-{G}_{y}^{i}$$

where ${G}_{x,L}^{i}$ and ${G}_{x,R}^{i}$ are gradient in left and right directions in ${i}^{{th}}$ row, respectively. These gradient values are calculated using modified Sobel gradient as follows.

6 $${G}_{x,L}^{i}=\left[\begin{array}{cc}-1 & 1\\ -2 & 2\\ -1 & 1\end{array}\right]\circ \left[\begin{array}{cc}{L}_{i-1} & {C}_{i-1}\\ {L}_{i} & {C}_{i}\\ {L}_{i+1} & {C}_{i+1}\end{array}\right],{G}_{x,R}^{i}=\left[\begin{array}{cc}1 & -1\\ 2 & -2\\ 1 & -1\end{array}\right]\circ \left[\begin{array}{cc}{C}_{i-1} & {R}_{i-1}\\ {C}_{i} & {R}_{i}\\ {C}_{i+1} & {R}_{i+1}\end{array}\right]$$

where ${C}_{i}$, ${L}_{i}$ and ${R}_{i}$ are the mean of contact frequencies in $i$^th^ stripe row and adjacent left/right background rows (width = 50 kb), respectively. The gradient in *y*-direction ${G}_{y}^{i}$ is calculated as:

7 $${G}_{y}^{i}=\left[\begin{array}{ccc}1 & 2 & 1\\ 0 & 0 & 0\\ -1 & -2 & -1\end{array}\right]\circ \left[\begin{array}{ccc}{L}_{i-1} & {C}_{i-1} & {R}_{i-1}\\ {L}_{i} & {C}_{i} & {R}_{i}\\ {L}_{i+1} & {C}_{i+1} & {R}_{i+1}\end{array}\right]$$

Based on the definition, negative stripiness represents low-quality stripes since the contrast between the stripe and its background is weaker than the pixel continuity along with the stripe domain. Thus, we recommend excluding the stripes with negative stripiness to construct a reliable set of stripes.

### Benchmarking analysis

For competitive benchmarking, we used Hi-C data of 72 h-activated B cell (GSE82144). Zebra stripe calls were directly obtained from the author of the original paper^13^. We processed these calls to merge the adjacent calls and connect each call to the diagonal line of the contact frequency matrix. To run domainClassifyR^12^, we used three R packages (domainClassifyR, misha and shaman). TAD coordinates, required for this program, were assessed using two Perl scripts named ‘*matrix2insulation.pl*’ and ‘*insulation2tads.pl*’ from the cworld-Dekker Github page^60^. All parameters were set as defaults. Then calls with forward or reverse *z*-score >5 were used in the analysis to have enough calls for comparison. Chromosight version 1.6.1 was installed and 5′- and 3-stripes were detected using the following command lines, respectively.

$$Chromosight\,detect\!-\!-\! pattern=stripe\_right\!-\!-\! threads=10\,cool\_file\,stripe\_right$$

$$Chromosight\,detect\!-\!-\!pattern=stripe\_left\!-\!-\!threads=10\,cool\_file\,stripe\_left$$

Here, we used the Hi-C map with a resolution of 5 kb. Then we removed redundant pixels indicating the same stripes. In other words, if two pixels are located on the same line, the one that was closer to the diagonal line was eliminated. StripeCaller, the Github implementation of Zebra, was also performed using default parameters. Here, additional information such as TADs and loops was not provided for a fair comparison. Similar to Zebra, adjacent calls from StripeCaller were merged and extended to diagonal line. Stripenn stripes were called from unnormalized, 5kb-resolution data since the observed signals in Zebra calls were detected from the raw Hi-C ligation counts^13^. Time and memory usage of each method were measured using ‘*time*’ with -*v* option (/usr/bin/time -v). This comparison was performed on a server computer of which CPU model was Intel ® Xeon® Gold 5115 CPU @ 2.40 GHz having 394.9 GB RAM.

1. Pileup plot analysis: In Fig. 1d, pileup plot of all stripes detected by each method was generated using coolpup.py program^61^ where ‘--local –rescale --unbalanced’ options were used. Similarly, in Supplementary Fig. 2d, the pileup plot of common and specific stripes from Zebra and StripeCaller was generated using the identical command line.
2. Common and unique stripes*:* For Zebra and Stripenn, which provide exact stripe coordinates, we compared stripiness and the average observed/expected contact frequency of common and unique stripes. We regarded two stripes were overlapping if they shared at least one genomic bin.

### Downsampling analysis

The FASTQ file of the Hi-C data of 72 h-activated B cell (GSE82144) was downloaded and processed in HiC-pro^62^ to obtain 212.8 million valid pairs. The downsampling of this dataset was performed to identify the minimum level of valid pairs to run Stripenn. Among 212.8 M valid pairs, 200 M, 175 M, 150 M, 125 M, 100 M, 75 M, 50 M, 25 M valid pairs were sampled using the *shuf* function. Each shuffled file was converted to.hic format using Juicer^18^ and then converted to.cool format using hic2cool version 0.8.3^63^. Stripes were predicted from each.cool file for chromosome 1 with different maximum pixel quantile parameters (0.9 to 0.995 with step 0.1), and the running time was measured. The number of threads was fixed to ten.

### Example datasets for chromatin conformation capture measurements

The example stripes in Supplementary Fig. 2a, b were from (1) Hi-C data of 30 hours-activated B cell (4DNFIOJNOH8U), (2) HiChIP data of DP thymocytes (GSE141847), (3) Micro-C of human foreskin fibroblast (4DNFIQXJQWD8) and Hi-C data of drosophila (4DNFIZ1ZVXC8). Each example was visualized using Juicebox^18,64^.

### Normalization of chromatin conformation measurement data

Square root vanilla coverage method^17^ was applied to normalize Hi-C of mouse 30 hours-activated B cell data^13^, CH12 B cell lymphoma cell line^13^, mouse DN3 T cell line, mouse Th1 cells, human CD4^+^ cells, and HiChIP data of DP thymocytes of C57BL/6J and NOD strains^31^. Square root vanilla coverage normalization (5103 stripes) performs better than Vanilla coverage (2,407 stripes), KR balancing normalization (4361 stripes) in terms of the number of stripes detected when applied to the micro-C of human foreskin fibroblasts.

### Compartment analysis

Principal component (PC) analysis was performed using HOMER^20^ with 50 kb resolution. The regions with positive and negative PC values were regarded as A and B compartments, respectively.

### Protein binding intensity heatmap

Our custom R code generated the protein binding intensity heatmaps in Supplementary Figs. 4g, S5b, d. The heatmap represents the protein binding intensity on a series of rescaled stripes. Each stripe domain was rescaled to 100 bins, and the binding intensities on the stripe domain were linearly interpolated. The padding bin sizes are 50 for 5′- and 3′-end of stripe, respectively. The graph on the top of the heatmap represents the median binding intensity of each of the 200 bins.

### Nomenclature of 5′- and 3′- stripes

5-stripes have anchors at 5′-end of the stripe domains, and vice versa.

### Gene set analysis

Metascape is an effective and efficient web portal designed for experimentalists^33^. To facilitate the Omics data analysis, it integrates functional enrichment, interactome, gene annotation, and membership search analysis from over 40 independent knowledge databases such as Gene Ontology^65,66^, MSigDB^67,68^, KEGG^69^, Reactome, etc. A prominent characteristic of the Metascape is the comparative analysis across multiple independent and orthogonal experiments. Taking advantage of this, we performed the comparative gene ontology analysis of the most highly expressed genes in non-stripy and stripy TAD of mouse DP thymocytes. Both input and analysis species were set as *Mus musculus*. In addition, gene set analyses for individual gene lists were performed for the most highly expressed genes in B and T cells (species: *mus musculus*) and those in conserved stripes and loops across humans and mice (species: *homo sapiens*).

### Stripiness/median *P*-value comparison between two datasets

1. *Two mouse strains*: The stripes from NOD and C57BL/6J DP thymocyte HiChIP data were compared. First, the stripes were searched from both 5 kb and 10 kb resolution data and then filtered based on median *P*-value < 0.1. Stripes from four datasets (NOD/5 kb, NOD/10 kb, C57BL/6J/5 kb, and C57BL6J/10 kb) were then merged as follows. First, stripes from the same strain but different resolution data were combined. For overlapping stripes, stripes with the largest stripiness were selected. Next, stripes from different strains were combined. For combined stripes, stripiness and median *P*-values were recalculated based on NOD and C57BL/6J HiChIP data with 5 kb resolution using Stripenn’s *score* function.
2. *B and T cells*: The stripes were predicted from the Hi-C measurements of CH12 B lymphoma cell lines and DN3 T cells, respectively, using Stripenn (median P-value < 0.05, 5 kb resolution). Similar to the two mouse strain comparison, the stripes from two datasets were merged, and then their stripiness and median *P*-values on each Hi-C data were estimated using Stripenn’s *score* function.

### Cell-type-specific stripes

In this study, the cell-type-specific stripes were defined as those with stripiness >2 in one condition and stripiness <0 in the other condition.

### Super-enhancer analysis

Using the previously reported strategy^49^, we estimated typical and super-enhancers of mouse DP thymocytes from the H3K27ac ChIP-seq of C57BL/6 J strain. Briefly, the H3K27ac peaks were merged for every genomic bin (12,500 bp) and then ranked. In Fig. 2b, the elbow of the plot represents the super-enhancers. The overlap between stripes and each genomic bin was tested using an R package called *GenomicRanges*^70^. The significance of the overlap between stripes and super-enhancer was estimated using the hypergeometric distribution.

### Loop detection

Loops from Hi-C measurements (5kb-resolution) of human CD4^+^ T cells and mouse Th1 cells were detected using Mustache version 1.2.0^51^, and FDR < 0.01 was used as the cutoff. Loops overlapping with stripe domains were excluded from the analysis.

### Lift genome annotation

The genome coordinates of the mouse (mm10) stripe anchors, defined as the +50 kb region from the stripe start site, were converted to human (hg38) coordinates using *LiftOver*^71^. The *minMatch* parameter was set as 0.1.

### Triangle heatmaps

Triangle heatmaps and corresponding tracks were generated by R package Sushi.

### Statistical analysis

Statistical significance was tested using two-sided Wilcoxon rank sum test. *P < 0.05; **P < 0.01; ***P < 0.001; ****P < 0.0001.

### Reporting summary

Further information on research design is available in the Nature Research Reporting Summary linked to this article.

## Supplementary information

Supplementary Information

Reporting Summary

## Data Availability

The data that support this study are available from the corresponding author upon reasonable request. The Hi-C data for DN3 T cells generated in this study have been deposited in the GEO:NCBI database under accession code GSM5388162. Publicly available data used in our study are B-cell Hi-C (30 h): 4DNFIOJNOH8U, B-cell Hi-C (72 h): GSE82144, T-cell HiChlP: GSE141847, HFF Micro-C: 4DNFIQXJQWD8, Drosophila Hi-C: 4DNFIZ1ZVXC8, B cell lymphoma Hi-C: 4DNFIASQYF5S, ChlP-seq for B-cell: 4DNES64LTQ68 (CTCF), 4DNESRQNIDVZ (Nipbl), 4DNESQ6W1U8J (Rad21), 4DNESC14YQV5 (Smc3), ChIP-seq and ATAC-seq for T-cell: GSE141853. Source data are provided with this paper.

## Code availability

The source code of Stripenn (version 1.1.65) is available on the author’s Github page (https://github.com/VahediLab/stripenn). The codes for data analysis are available on this Github page (https://github.com/VahediLab/stripenn-data-code).
