# Supplementary material for: Stripenn detects architectural stripes from chromatin conformation data using computer vision
Source: Nat Commun. 2022 Mar 24;13:1602. doi: 10.1038/s41467-022-29258-9 (PMC8948182; doi:10.1038/s41467-022-29258-9)
Supplement: Supplementary file 1 — Supplementary Information [file 41467_2022_29258_MOESM1_ESM.pdf]

Supplementary file for the study:

Stripenn detects architectural stripes from chromatin conformation data using computer vision

Sora Yoon[1,2,3,4], Aditi Chandra[1,2,3,4], Golnaz Vahedi[1,2,3,4,5*]

[1]Department of Genetics, [2]Institute for Immunology, [3]Epigenetics Institute, [4]Institute for Diabetes, Obesity and Metabolism, [5]Abramson Family Cancer Research Institute, University of Pennsylvania Perelman School of Medicine, Philadelphia, PA 19104, USA

Corresponding author: vahedi@pennmedicine.upenn.edu

Supplementary Figure 1

a

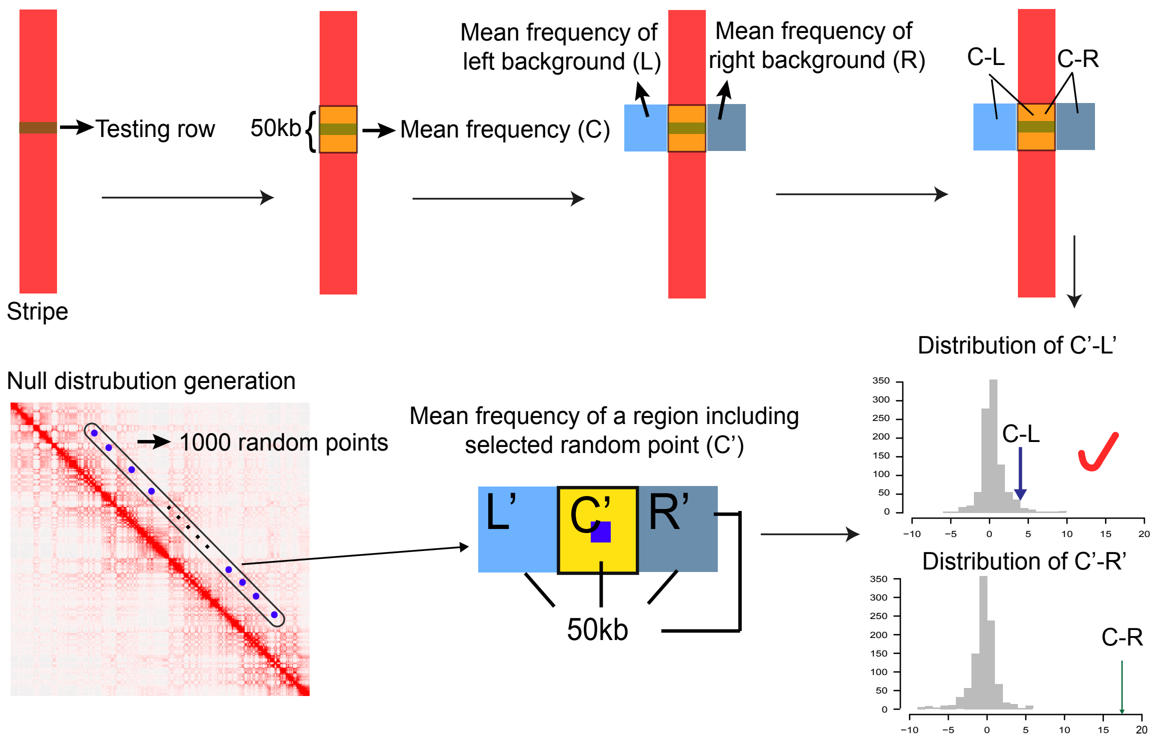

b

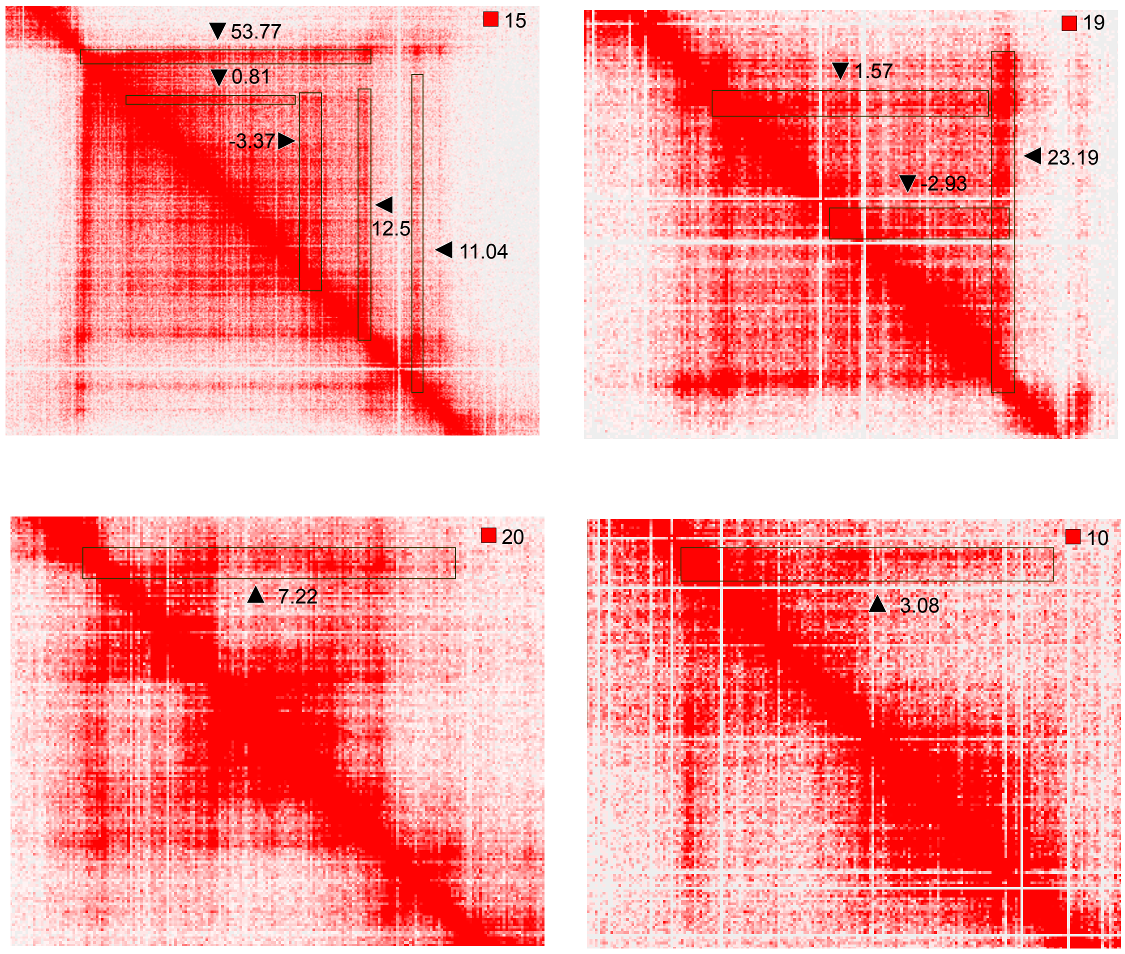

Supplementary Figure 1. Median P-value estimation in Stripenn and example stripes with various stripiness

(a) Description of how to estimate the *P*-value of an individual row in a stripe. For a testing row (marked green), the mean contact frequency (*C*) is calculated for N rows (yellow) corresponding to 50kb, including the testing row at the center. The mean frequency of left (L, sky blue) and right (R, blue) adjacent backgrounds are also calculated. Here, the background size is N x N bins. Then, the significance of *C-L* and *C-R* are estimated based on the null distribution of contact frequency difference. To construct null distribution, 1000 random points were selected from which bin distance is identical to that of stripe anchor and testing row. Here, null values for center (C'), left (L') and right (R') mean contact frequencies are calculated from N x N matrices, respectively. The significance of C-L and C-R are estimated based on null distribution, and a less significant value is selected. (b) Example stripes with various stripiness. Contrast with adjacent pixels, pixel continuity, and median pixel intensity determine the stripiness.

Supplementary Figure 2

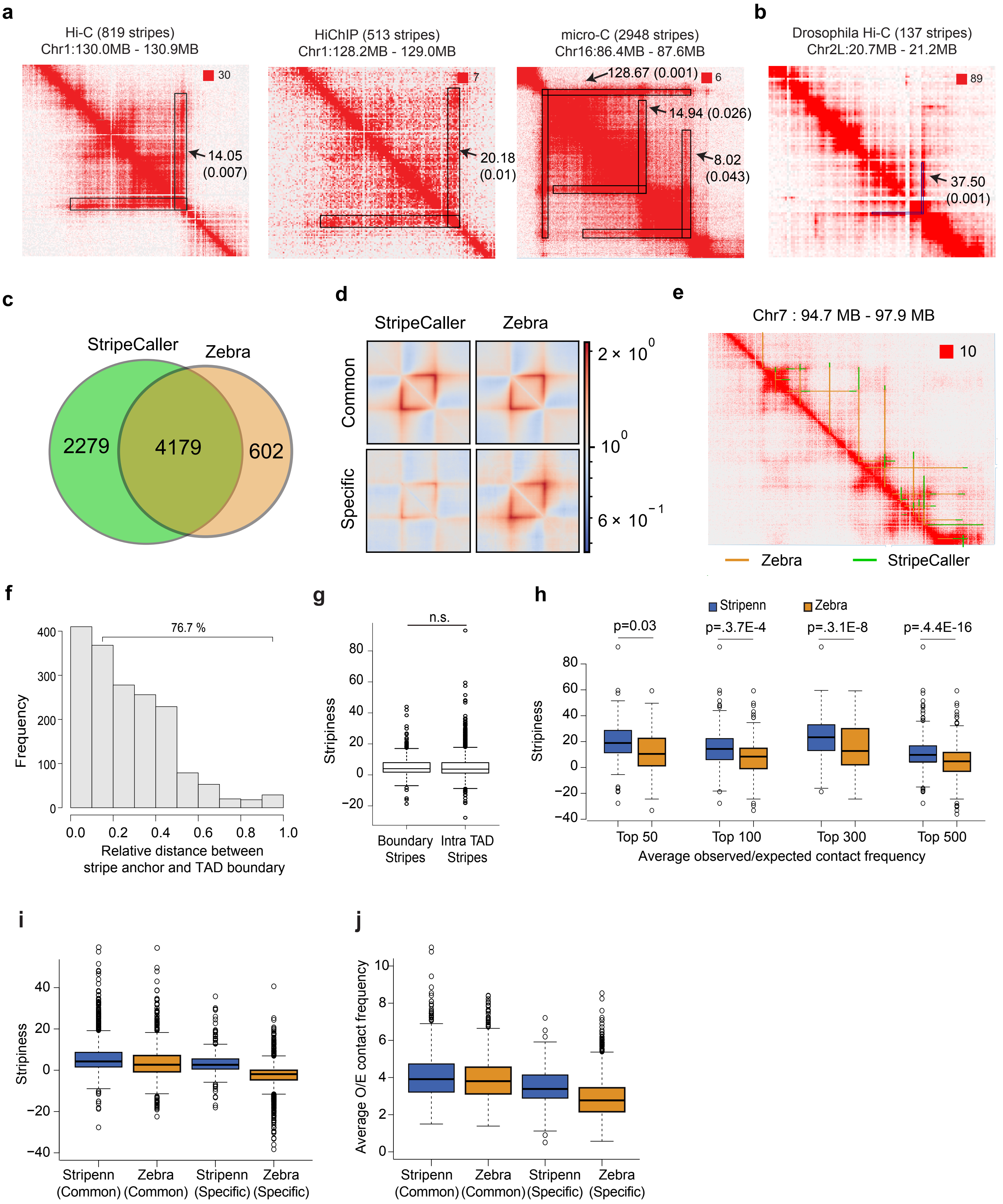

Supplementary Figure 2. Stripenn detects stripes from various chromatin conformation capture data and outperforms Zebra. (a) Example stripe calls (marked black border) from Hi-C (activated B-cell), HiChIP (DP T-cell) and Micro-C (human foreskin fibroblast) data. Stripenn detected 819, 513 and 2,948 stripes from three data, respectively (median *P*-value < 0.05). Stripiness and median *P*-values (in the parenthesis) are shown for each stripe. (b) Example stripe call with stripiness and median *P*-value (in the parenthesis) from Hi-C data in *Drosophila* which yielded 137 stripes (median *P*-value < 0.05). (c) Venn diagram showing the number of stripe calls detected by StripeCaller (left) and Zebra (right). (d) Pileup plots of common and uniquely detected stripes from StripeCaller (left) and Zebra (right) (e) The example stripe calls from Zebra (orange) and StripeCaller (green). (f) Histogram demonstrates the relative distance between stripe anchors and TAD boundaries obtained from activated B-cell Hi-C data. For 5'-stripe (3'-stripe), the relative distance was defined as the distance between the stripe anchor and 5'-end (3'-end) of TAD boundary where the anchor was included divided by the TAD size. More than 70% of stripes showed a relative distance>0.1. (g) Stripiness comparison between boundary stripes (relative distance < 0.1, N=410) and intra-TAD stripes (relative distance >= 0.1, N=1,348) from the activated B cell Hi-C data. Data are shown as boxplots (centre, median; box limits, upper (75th) and lower (25th) percentiles; whiskers,1.5×interquartile range; points, outliers) (h) The stripiness was compared between Stripenn and Zebra stripes for the top *N* average observed/expected (O/E) contact frequency (*N*=50, 100, 300, 500). Here, activated B cell Hi-C data was used. Data are shown as boxplot of which format is identical to (g). (i) Stripiness comparison between Stripenn (blue) and Zebra (orange). The stripiness distribution of overlapping stripes was different between Stripenn and Zebra because the stripe coordinates are not identical between the two methods. Stripiness comparison of the unique stripes are shown in Figure 1e. Data are shown as boxplot of which format is identical to (g). (j) The average observed/expected contact frequency of Stripenn and Zebra stripes. Zebra-specific stripes were weaker than those of Stripenn overall. Data are shown as boxplot of which format is identical to (g). Statistical significance was tested using two-sided Wilcoxon rank sum test.

Supplementary Figure 3

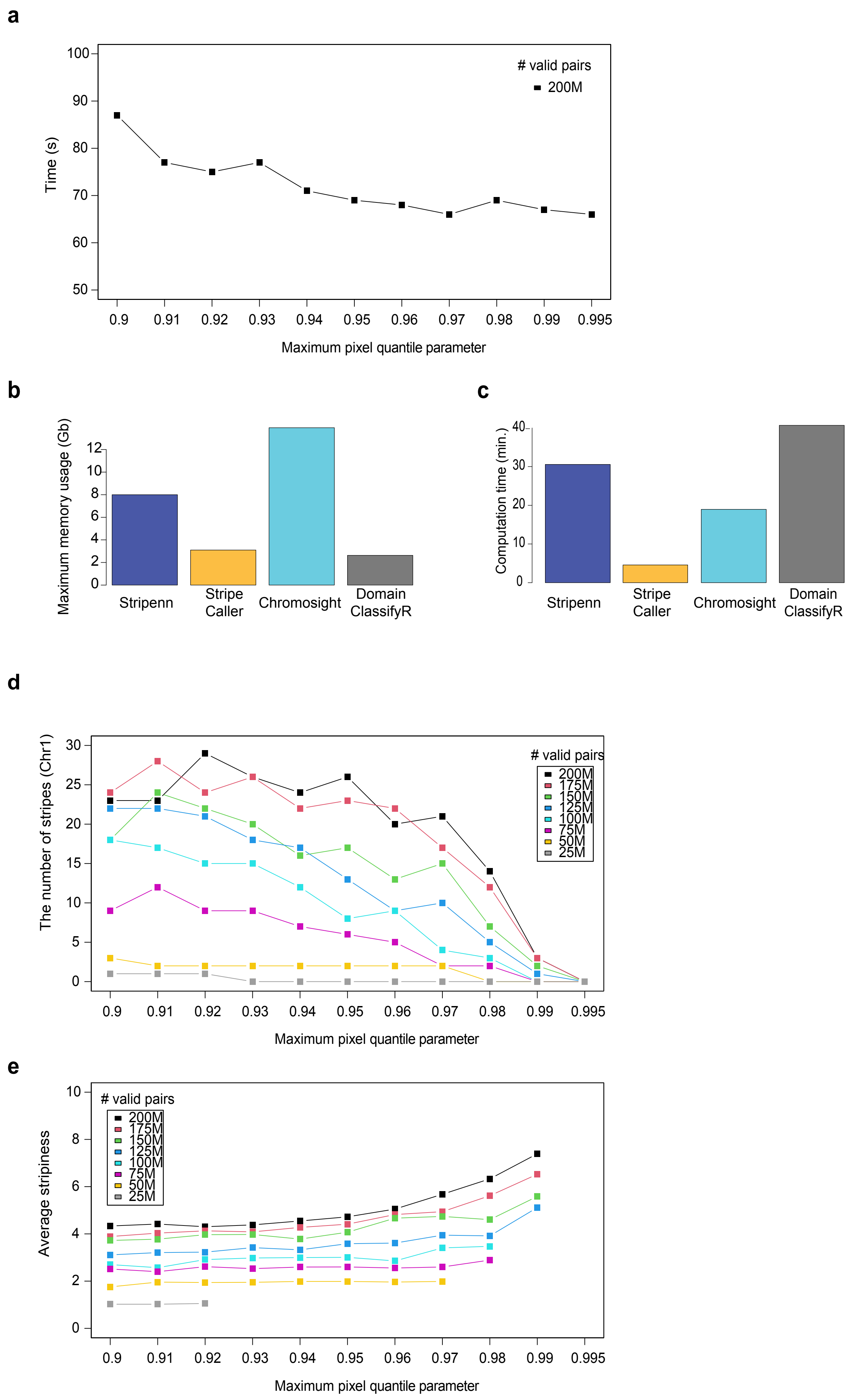

Supplementary Figure 3. Downsampling analysis

(a) The computation time of Stripenn detecting stripes from the identical datasets. The number of valid pairs was fixed as 200 M. (b) The maximum memory usage and (c) computation time of Stripenn, StripeCaller, Chromosight and DomainClassifyR. Here, stripes from all chromosomes were detected since Chromosight does not have the option to choose chromosomes. For DomainClassifyR, the time taken for obtaining Hi-C contact frequency data and running domainClassifyR were added. (d-e) The number (d) and the average stripiness (e) of stripes detected from mouse B cell chromosome 1 using Stripenn for different numbers of valid pairs and maximum pixel percentile parameter.

Supplementary Figure 4

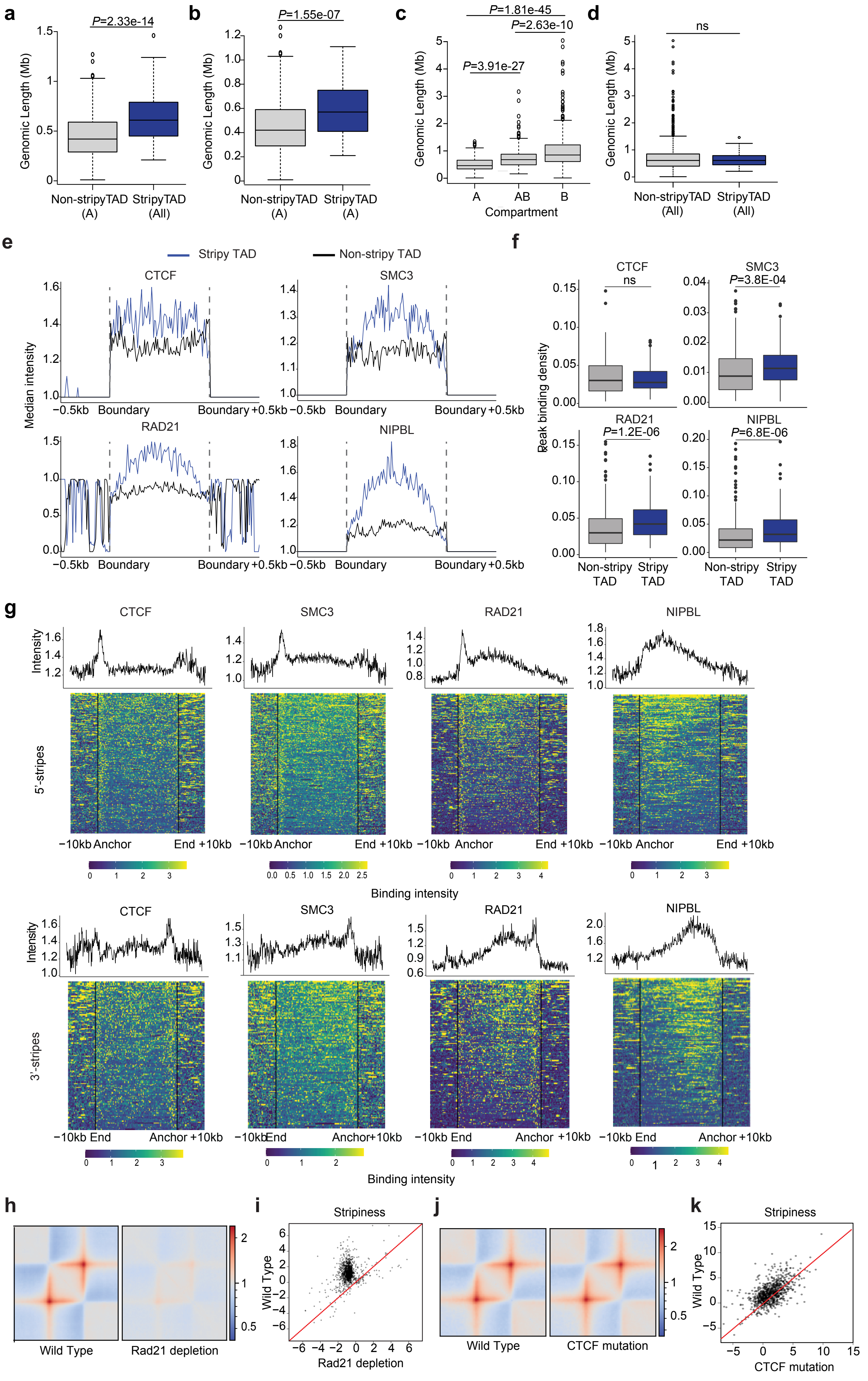

Supplementary Figure 4. Comparison between the stripy and non-stripy TADs using the Hi-C data.

(a-d) Genomic size distribution of (a) non-stripy TADs in only A compartment (N=286) and all stripy TADs (N=155), (b) non-stripy and stripy TADs, both included in only A compartment (N=286 and N=101, respectively), (c) TADs only in A (N=571), across A and B (AB, N=404), and only in B compartment (N=308), and (d) all non-stripy TADs (N=286) and stripy TADs (N=155). For (a-d), . Data are shown as boxplots (centre, median; box limits, upper (75th) and lower (25th) percentiles; whiskers, 1.5 × interquartile range; points, outliers). (e) Median binding intensity of CTCF, SMC3, RAD21, and NIPBL on rescaled stripy TAD (blue) and non-stripy TAD (black). Dashed lines represent the TAD boundary. (f) The structural protein peak binding density in stripy (blue) and non-stripy (gray) TAD. For each TAD, the genomic size of total protein binding sites at the peak was divided by the TAD size. (g) Distribution of CTCF, SMC3, RAD21 and NIPBL on rescaled 5'- (up) and 3'-stripes (down). The profiles represent the median binding intensity of each protein. Black lines in the heatmaps are the stripe boundaries. The format of boxplots is identical to those in (a-d). (h) Pile-up plots of stripes detected in wild-type (left) and Rad21-depleted HCT116 cell lines (right). (i) Stripiness comparison between wild-type and Rad21-depleted HCT116 cell lines. (j) Pile-up plots of stripes detected in wild-type (left) and CTCF-mutated (right) mouse B cell lymphoma cells (CH12). (k) Stripiness comparison between wild-type and CTCF-mutated mouse B cell lymphoma cells (CH12). Statistical significance was tested using two-sided Wilcoxon rank sum test.

Supplementary Figure 5

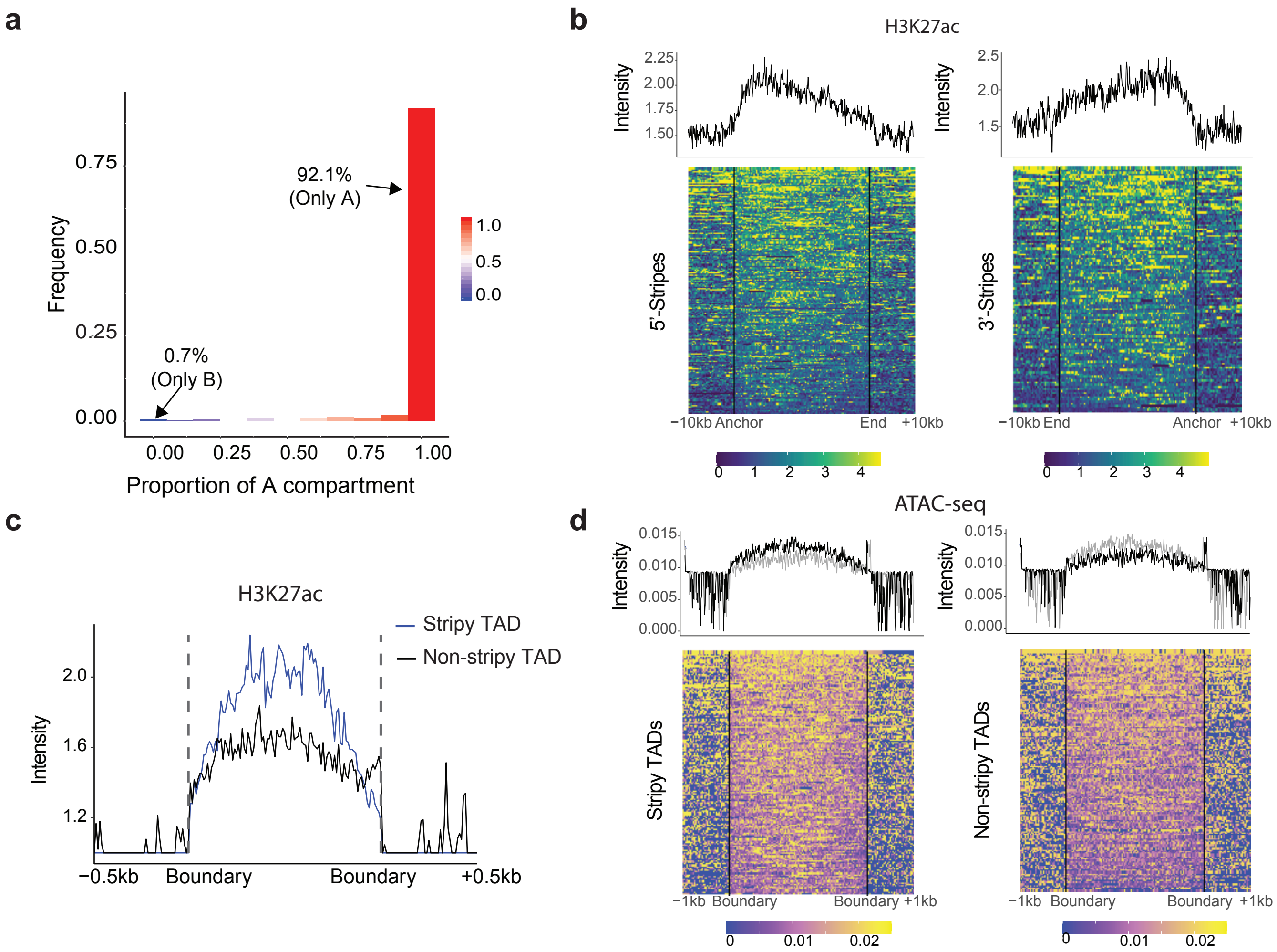

Supplementary Figure 5. Stripy TADs are epigenetically more active and accessible.

(a) The proportion of A compartment within stripe domains from DP thymocytes HiChIP data. 92.1% of stripes are solely in A compartment. (b) The binding intensity of histone mark (H3K27ac) along with the rescaled stripes from HiChIP data. Left and right plots correspond to 5'- and 3'-stripes, respectively. Yellow pixels represent high intensity. (c) The median binding intensity of H3K27ac on the rescaled stripy (blue) and non-stripy (black) TADs. The dashed line represents the TAD boundaries. (d) Comparison of DNA accessibility between stripy TADs (left) and non-stripy TADs (right). The heatmaps represent the degree of DNA accessibility. Solid black lines in the heatmaps show TAD boundaries. Blackline in the upper profile is the median intensity of stripy (left) and non-stripy (right) TADs. The gray line is the median intensity of non-stripy (left) and stripy (right) TADs, added for the comparison.

Supplementary Figure 6

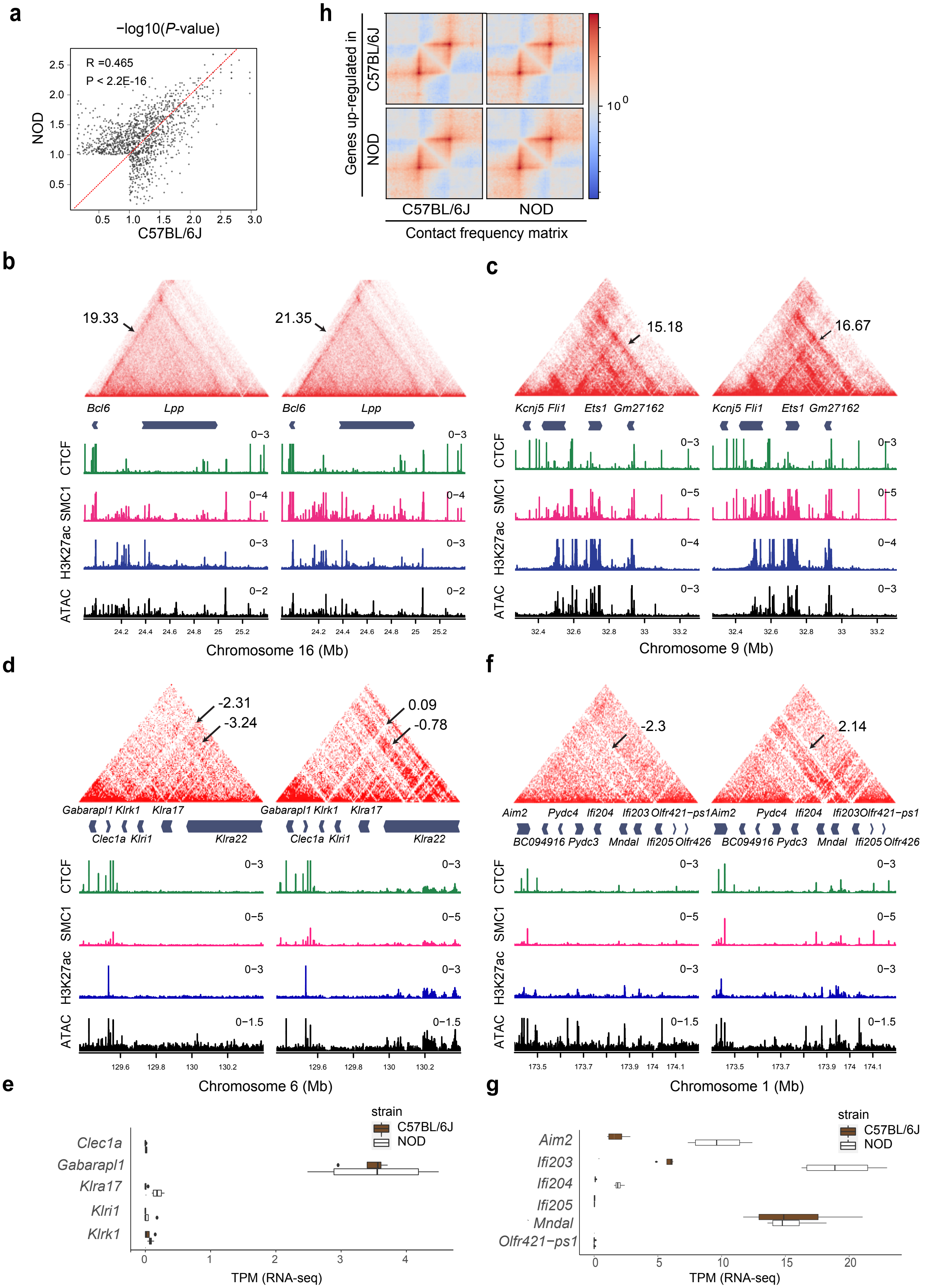

Supplementary Figure 6. The effect of natural genetic variation on stripe. (a) Stripes from DP thymocytes HiChIP of NOD and C57BL/6J (median *P*-value < 0.1) were merged, and the median *P*-value was recalculated based on HiChIP measurements of each strain. Median *P*-values from two strains show significant correlation (Pearson correlation test *P*-value < 2.2e-16). (b-c) Stripes and binding intensity of CTCF, SMC1, H3K27ac and Tn5 transposase at (b) *Bcl6* and (c) *Ets1* loci. Stripes are marked with an arrow. (d,f) Differential stripe between NOD and C57BL/6J at (d) killer cell lectin-like receptor (KLR) gene family loci and (f) *Aim2* loci. (e,g) Expression level of genes nearby (e) KLR family and (h) *Aim2* in C57BL/6J (brown, N=4 biological replicates) and NOD (white, N=4 biological replicates). Data are shown as boxplots (centre, median; box limits, upper (75th) and lower (25th) percentiles; whiskers, maximum and minumum) (h) Pileup plot of stripes harboring the differentially expressed genes in C57BL/6J (upper) and NOD (bottom) strains. No dramatic change was observed between the stripes from T cells of C57BL/6J and NOD on average.

Supplementary Figure 7

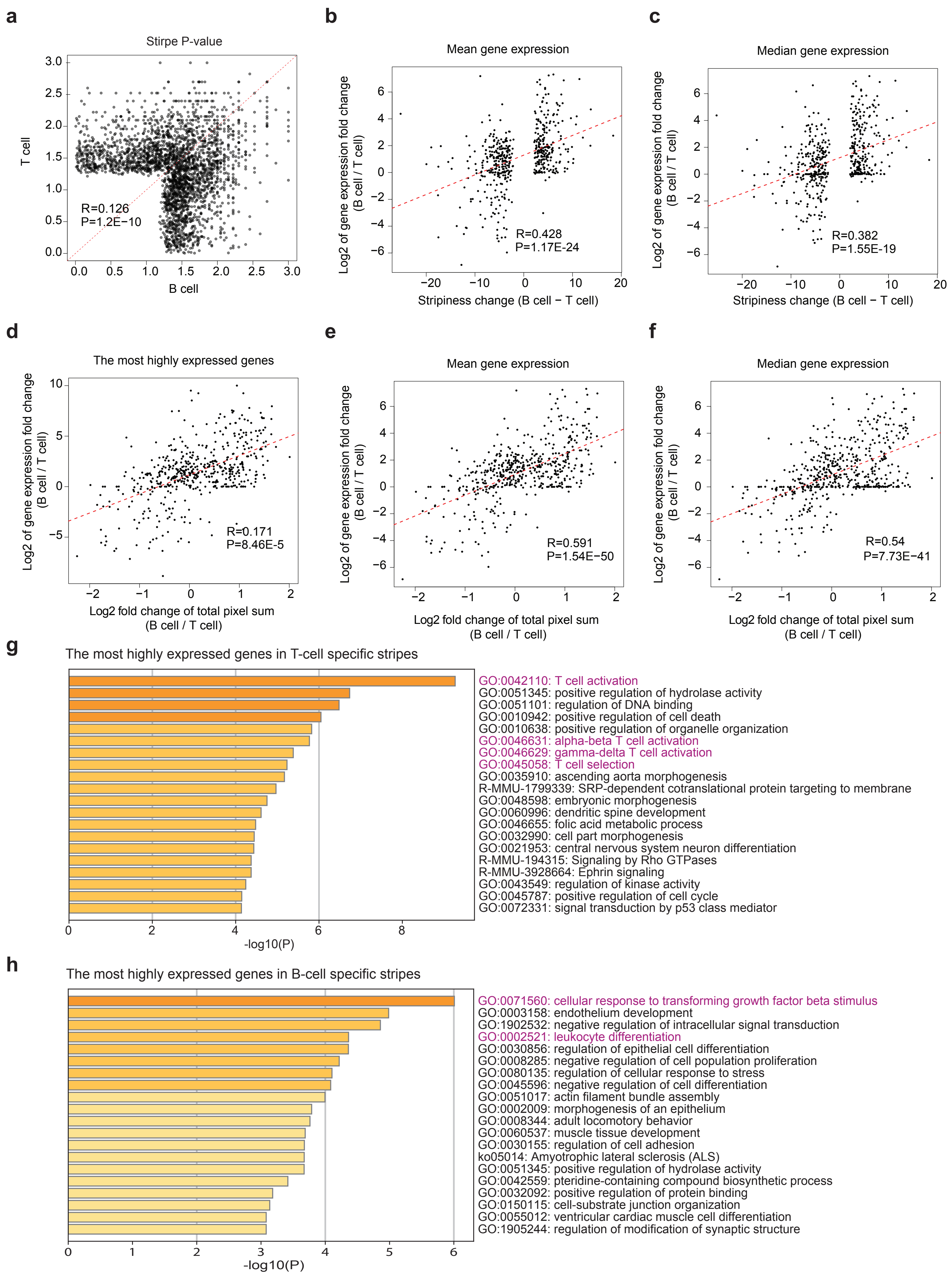

Supplementary Figure 7. Stripiness and the stripe intensity changes are correlated with the gene expression change.

(a) Stripes from Hi-C data of CH12 B lymphoma cell lines and DN3 T cells (median *P*-value < 0.05) were merged, and the median *P*-value was recalculated based on contact frequency in each cell type. Median *P*-values from two cell types showed a significant but much-reduced correlation compared with the analysis between two mouse strains (Two-sided Pearson correlation test *P*-value < 1.2E-10). (b-c) The relationship between stripiness change and expression level changes. For each stripe, (b) the average and (c) the median gene expression were used, respectively. (d-f) The relationship between the changes in the total sum of the observed/expected pixels and the gene expression changes was measured. For each stripe, (d) the most highly expressed, (e) the average, and (f) the median gene expression changes were used, respectively. For (b-f), two-sided Pearson correlation test was performed. (g-h) Gene ontology analysis of the most highly expressed genes in (g) T and (h) B cell-specific stripes defined in figure 3f. Here, Metascape was used where hypergeometric test and Benjamini-Hochberg correction were applied.
