# Supplementary material for: Stripenn detects architectural stripes from chromatin conformation data using computer vision
Source: Nat Commun. 2022 Mar 24;13:1602. doi: 10.1038/s41467-022-29258-9 (PMC8948182; doi:10.1038/s41467-022-29258-9)
Supplement: Supplementary file 2 — Reporting Summary [file 41467_2022_29258_MOESM2_ESM.pdf]

nature portfolio

Corresponding author(s): Golnaz Vahedi, PhD

Last updated by author(s): Feb 23, 2022

Reporting Summary

Nature Portfolio wishes to improve the reproducibility of the work that we publish. This form provides structure for consistency and transparency in reporting. For further information on Nature Portfolio policies, see our Editorial Policies and the Editorial Policy Checklist.

Statistics

For all statistical analyses, confirm that the following items are present in the figure legend, table legend, main text, or Methods section.

n/a	Confirmed	
☐	☒	The exact sample size (*n*) for each experimental group/condition, given as a discrete number and unit of measurement
☐	☒	A statement on whether measurements were taken from distinct samples or whether the same sample was measured repeatedly
☐	☒	The statistical test(s) used AND whether they are one- or two-sided<br>*Only common tests should be described solely by name; describe more complex techniques in the Methods section.*
☒	☐	A description of all covariates tested
☐	☒	A description of any assumptions or corrections, such as tests of normality and adjustment for multiple comparisons
☐	☒	A full description of the statistical parameters including central tendency (e.g. means) or other basic estimates (e.g. regression coefficient) AND variation (e.g. standard deviation) or associated estimates of uncertainty (e.g. confidence intervals)
☐	☒	For null hypothesis testing, the test statistic (e.g. *F*, *t*, *r*) with confidence intervals, effect sizes, degrees of freedom and *P* value noted<br>*Give P values as exact values whenever suitable.*
☒	☐	For Bayesian analysis, information on the choice of priors and Markov chain Monte Carlo settings
☒	☐	For hierarchical and complex designs, identification of the appropriate level for tests and full reporting of outcomes
☒	☐	Estimates of effect sizes (e.g. Cohen's *d*, Pearson's *r*), indicating how they were calculated

Our web collection on statistics for biologists contains articles on many of the points above.

Software and code

Policy information about availability of computer code

Data collection	1. Stripe calls from Zebra were directly obtained from the author of the original paper (Vian et al., 2018)
Data analysis	2. Python 3.8 was used for Stripenn development.<br>3. Stripes were extracted using Stripenn (version 1.1.50 and 1.1.65)<br>4. R version 3.6.2 was used to generate Venn diagram (VennDiagram package version 1.6.20), box plot, scatter plot, histogram, line plot and heatmap (ggplot2 version 3.3.3)<br>5. Juicebox (version 1.11.08) was used to visualize the 3D genome interaction image. (Durand, Cell syst. 2016)<br>6. TADs were extracted from chromatin conformation capture data by (1) converting to matrix using juicer_tools dump function and then (2) calculating insulation score using matrix2insulation.pl and (3) finding local minimum using insulation2tad.pl. matrix2insulation.pl and insulation2tad.pl are available from cworld-dekker Github page (https://github.com/dekkerlab/cworld-dekker)<br>7. DomainClassifyR (version 0.0.0.9000) was used to calculate stripe/loop score for each TAD. (Barrington et al., Nature Communications, 2019)<br>8. Coolpup.py (version 0.9.1) was used to generate pileup plots (Flyamer, Bioinformatics, 2020).<br>9. Metascape was used for gene set enrichment analysis. (https://metascape.org/gp/index.html#, Zhou et al., Nature Communications, 2019)<br>10. Sushi R package was used to visualize chromatin conformation capture data and ChIP-seq track (1.28.0)<br>11. Enhancer analysis followed the strategy in (Vahedi et al., 2015)<br>12. IGV (version 2.9.4) was used to visualize the CTCF, H3K27ac and RNA-seq track in Figure 2f.<br>13. Loops from Hi-C of human CD4+ T cells and mouse Th1 cells were detected using Mustache (version 1.2.0). FDR< 0.01 was used as cutoff.<br>14. Liftover (Hinrichs et al., 2006) was used to convert the coordinates of loops/stripes in mouse to those of human.

For manuscripts utilizing custom algorithms or software that are central to the research but not yet described in published literature, software must be made available to editors and reviewers. We strongly encourage code deposition in a community repository (e.g. GitHub). See the Nature Portfolio guidelines for submitting code & software for further information.

Data

Policy information about availability of data

All manuscripts must include a data availability statement. This statement should provide the following information, where applicable:

- Accession codes, unique identifiers, or web links for publicly available datasets
- A description of any restrictions on data availability
- For clinical datasets or third party data, please ensure that the statement adheres to our policy

The HiC data generated in this study have been deposited in the GEO:NCBI database under accession code GSE178348. Publicly available data used in our study are B-cell Hi-C (30 hrs): 4DNFIOJNOH8U , B-cell Hi-C (72 hrs): GSE82144, T-cell HiChIP: GSE141847, HFF Micro-C: 4DNFIQXJQWD8, Drosophila Hi-C: 4DNFIZ1ZVXC8, B cell lymphoma Hi-C: 4DNFIASQYF5S , ChIP-seq for B-cell: 4DNES64LTQ68 (CTCF), 4DNESRQNIDVZ (Nipbl), 4DNESQ6W1U8J (Rad21), 4DNESC14YQV5 (Smc3) , ChIP-seq and ATAC-seq for T-cell: GSE141853.

Field-specific reporting

Please select the one below that is the best fit for your research. If you are not sure, read the appropriate sections before making your selection.

☒ Life sciences ☐ Behavioural & social sciences ☐ Ecological, evolutionary & environmental sciences

For a reference copy of the document with all sections, see nature.com/documents/nr-reporting-summary-flat.pdf

Life sciences study design

All studies must disclose on these points even when the disclosure is negative.

Sample size	Sample size specifics are provided in Figure legends
Data exclusions	No samples were excluded.
Replication	Biological replicates were used for DESeq analysis.
Randomization	Randomization was not carried out.
Blinding	Blinding was not carried out because genomic approaches are generally unbiased

Reporting for specific materials, systems and methods

We require information from authors about some types of materials, experimental systems and methods used in many studies. Here, indicate whether each material, system or method listed is relevant to your study. If you are not sure if a list item applies to your research, read the appropriate section before selecting a response.

Materials & experimental systems

n/a	Involved in the study	
☐	☒	Antibodies
☐	☒	Eukaryotic cell lines
☒	☐	Palaeontology and archaeology
☐	☒	Animals and other organisms
☐	☒	Human research participants
☒	☐	Clinical data
☒	☐	Dual use research of concern

Methods

n/a	Involved in the study	
☒	☐	ChIP-seq
☒	☐	Flow cytometry
☒	☐	MRI-based neuroimaging

Antibodies

Antibodies used	anti-human CD3 (BD biosciences, cat#565120), anti-human CD14 (BioLegend, cat#301842), anti-human CD19 (BioLegend, cat#302205), anti-human CD8 (BioLegend, cat#301040), anti-human CD4 (BioLegend, cat#317411)
Validation	Antibodies were purchased from certified commercial sources, each of which validated antibody quality by probing extracts from different cell types showing the correct size protein band.

Eukaryotic cell lines

Policy information about cell lines

Cell line source(s)	Scid.adh cell line was provided from Warren Pear lab at UPENN
Authentication	NA
Mycoplasma contamination	NA
Commonly misidentified lines (See ICLAC register)	NA

Animals and other organisms

Policy information about studies involving animals; ARRIVE guidelines recommended for reporting animal research

Laboratory animals	C57BL6/J mouse strain
Wild animals	NA
Field-collected samples	NA
Ethics oversight	All animal work was in accordance with the Institutional Animal Care and Use Committee of the University of Pennsylvania in accordance with NIH guidelines.

Note that full information on the approval of the study protocol must also be provided in the manuscript.

Human research participants

Policy information about studies involving human research participants

Population characteristics	Only one human sample from HPAP used for Hi-C profiling.
Recruitment	Pancreatic islets were procured by the HPAP consortium (RRID:SCR_016202; https://hpap.pmacs.upenn.edu), part of the Human Islet Research Network (https://hirnetwork.org/), with approval from the University of Florida Institutional Review Board (IRB # 201600029) and the United Network for Organ Sharing (UNOS).
Ethics oversight	Pancreatic islets were procured by the HPAP consortium under the Human Islet Research Network with approval.

Note that full information on the approval of the study protocol must also be provided in the manuscript.
